# Differences Between Robin and Neumann Eigenvalues

**DOI:** 10.1007/s00220-021-04248-y

**Published:** 2021-11-02

**Authors:** Zeév Rudnick, Igor Wigman, Nadav Yesha

**Affiliations:** 1 School of Mathematical Sciences, Tel Aviv University, 69978 Tel Aviv, Israel; 2 Department of Mathematics, King’s College London, London, UK; 3 Department of Mathematics, University of Haifa, 3498838 Haifa, Israel

## Abstract

Let $\Omega {\subset } {\mathbb {R}}^2$ be a bounded planar domain, with piecewise smooth boundary $\partial \Omega$. For $\sigma >0$, we consider the Robin boundary value problem

$$\begin{aligned} -\Delta f =\lambda f, \qquad \frac{\partial f}{\partial n} + \sigma f = 0 \text{ on } \partial \Omega \end{aligned}$$

where $\frac{\partial f}{\partial n}$ is the derivative in the direction of the outward pointing normal to $\partial \Omega$. Let $0<\lambda ^\sigma _0\le \lambda ^\sigma _1\le \ldots$ be the corresponding eigenvalues. The purpose of this paper is to study the Robin–Neumann gaps

$$\begin{aligned} d_n(\sigma ):=\lambda _n^\sigma -\lambda _n^0 . \end{aligned}$$

For a wide class of planar domains we show that there is a limiting mean value, equal to $2{\text {length}}(\partial \Omega )/{\text {area}}(\Omega )\cdot \sigma$ and in the smooth case, give an upper bound of $d_n(\sigma )\le C(\Omega ) n^{1/3}\sigma$ and a uniform lower bound. For ergodic billiards we show that along a density-one subsequence, the gaps converge to the mean value. We obtain further properties for rectangles, where we have a uniform upper bound, and for disks, where we improve the general upper bound.

## Statement of Results

Let $\Omega {\subset } {\mathbb {R}}^2$ be a bounded planar domain, with piecewise smooth boundary $\partial \Omega$. For $\sigma \ge 0$, we consider the Robin boundary value problem

$$\begin{aligned} -\Delta f =\lambda f \;\mathrm{on}\;\Omega , \quad \frac{\partial f}{\partial n} + \sigma f = 0 \text{ on } \partial \Omega \end{aligned}$$

where $\frac{\partial f}{\partial n}$ is the derivative in the direction of the outward pointing normal to $\partial \Omega$. The case $\sigma =0$ is the Neumann boundary condition, and we use $\sigma =\infty$ as a shorthand for the Dirichlet boundary condition $f|_{\partial \Omega }=0$.

Robin boundary conditions are used in heat conductance theory to interpolate between a perfectly insulating boundary, described by Neumann boundary conditions $\sigma =0$, and a temperature fixing boundary, described by Dirichlet boundary conditions corresponding to $\sigma =+\infty$. To date, most studies concentrated on the first few Robin eigenvalues, with applications in shape optimization and related isoperimetric inequalities and asymptotics of the first eigenvalues (see [5]). Our goal is very different, aiming to study the difference between high-lying Robin and Neumann eigenvalues. There are very few studies addressing this in the literature, except for [4, 32] which aim at different goals.

We will take the Robin condition for a fixed and positive $\sigma >0$, when all eigenvalues are positive, one excuse being that a negative Robin parameter gives non-physical boundary conditions for the heat equation, with heat flowing from cold to hot; see however [17] for a model where negative $\sigma$ is of interest, in particular $\sigma \rightarrow -\infty$ [10, 15, 21, 22]. Let $0<\lambda ^\sigma _0\le \lambda ^\sigma _1\le \ldots$ be the corresponding eigenvalues. The Robin spectrum always lies between the Neumann and Dirichlet spectra (Dirichlet–Neumann bracketing) [5] :

1.1 $$\begin{aligned} \lambda _n^0< \lambda _n^\sigma < \lambda _n^\infty . \end{aligned}$$

We define the Robin–Neumann difference (RN gaps) as

$$\begin{aligned} d_n(\sigma ):=\lambda _n^\sigma -\lambda _n^0 \end{aligned}$$

and study several of their properties. See Sect. 2 for some numerical experiments. This seems to be a novel subject, and the only related study that we are aware of is the very recent work of Rivière and Royer [28], which addresses the RN gaps for quantum star graphs.

### The mean value

The first result concerns the mean value of the gaps:

#### Theorem 1.1

Let $\Omega {\subset } {\mathbb {R}}^2$ be a bounded, piecewise smooth domain. Then the mean value of the RN gaps exists, and equals

$$\begin{aligned} \lim _{N\rightarrow \infty } \frac{1}{N} \sum _{n=1}^N d_n(\sigma ) = \frac{2{\text {length}}(\partial \Omega )}{{\text {area}}(\Omega )}\cdot \sigma . \end{aligned}$$

Since the differences $d_n(\sigma )> 0$ are positive, we deduce by Chebyshev’s inequality:

#### Corollary 1.2

Let $\Omega$ be a bounded, piecewise smooth domain. Fix $\sigma >0$. Let $\Phi (n)\rightarrow \infty$ be a function tending to infinity (arbitrarily slowly). Then for almost all *n*’s, $d_n(\sigma ) \le \Phi (n)$ in the sense that

$$\begin{aligned} \#\{n\le N : d_n(\sigma )> \Phi (n) \} \ll \frac{N}{\Phi (N)} . \end{aligned}$$

### A lower bound

Recall that a domain $\Omega$ is “star-shaped with respect to a point $x\in \Omega$" if the segment between *x* and every other point of $\Omega$ lies inside the domain; so convex means star-shaped with respect to any point; “star-shaped" just means that there is some *x* so that it is star-shaped with respect to *x*.

#### Theorem 1.3

Let $\Omega {\subset } {\mathbb {R}}^2$ be a bounded star-shaped planar domain with smooth boundary. Then the Robin–Neumann differences are uniformly bounded below: For all $\sigma >0$, $\exists C= C(\Omega ,\sigma )>0$ so that

$$\begin{aligned} d_n(\sigma ) \ge C . \end{aligned}$$

Note that for quantum star graphs, this lower bound fails [28].

### A general upper bound

We give a quantitative upper bound:

#### Theorem 1.4

Assume that $\Omega$ has a smooth boundary. Then $\exists C= C(\Omega )>0$ so that for all $\sigma >0$,

$$\begin{aligned} d_n(\sigma )\le C (\lambda _n^\infty )^{1/3} \sigma . \end{aligned}$$

While quite poor, it is the best individual bound that we have in general. Below, we will indicate how to improve it in special cases.

#### Question 1.5

Are there planar domains where the differences $d_n(\sigma )$ are **unbounded**?

We believe that this happens in several cases, e.g. the disk, but at present can only show this for the hemisphere [30], which is not a planar domain.

### Ergodic billiards

To a piecewise smooth planar domain one associates a billiard dynamics. When this dynamics is ergodic, as for the stadium billiard (see Fig. 2), we can improve on Corollary 1.2:

#### Theorem 1.6

Let $\Omega {\subset } {\mathbb {R}}^2$ be a bounded, piecewise smooth domain. Assume that the billiard dynamics associated to $\Omega$ is ergodic. Then for every $\sigma >0$, there is a sub-sequence $\mathcal N={\mathcal {N}}_\sigma {\subset } {\mathbb {N}}$ of density one so that along that subsequence,

$$\begin{aligned} \lim _{\begin{array}{c} n\rightarrow \infty \\ n\in {\mathcal {N}} \end{array}}d_n(\sigma )= \frac{2{\text {length}}(\partial \Omega )}{{\text {area}}(\Omega )}\cdot \sigma . \end{aligned}$$

If the billiard dynamics is uniformly hyperbolic, we expect that more is true, that *all* the gaps converge to the mean.

A key ingredient in the proofs of the above results is that they can be connected to $L^2$ restriction estimates for eigenfunctions on the boundary via a variational formula for the gaps (Lemma 3.1)

$$\begin{aligned} d_n(\sigma ) = \int _0^\sigma \left( \int _{\partial \Omega } |u_{n,\tau }|^2 ds \right) d\tau \end{aligned}$$

where $u_{n,\tau }$ is any $L^2(\Omega )$-normalized eigenfunction associated with $\lambda _n^\tau$.

### Generalizations

Most of the above results easily extend to higher dimensions: The upper bound (Theorem 1.4), the mean value result (Theorem 1.1), and the almost sure convergence for ergodic billiards (Theorem 1.6). At this stage our proof of the lower bound (Theorem 1.3) is restricted to dimension 2.

In Sect. 7 we discuss extensions of the above results to the case of variable boundary conditions $\sigma : \partial \Omega \rightarrow {\mathbb {R}}$.

### Rectangles

For the special case of rectangles, we show that the RN gaps are bounded:

#### Theorem 1.7

Let $\Omega$ be a rectangle. Then for every $\sigma >0$ there is some $C_\Omega (\sigma )>0$ so that for all *n*,

$$\begin{aligned} d_n(\sigma ) \le C_\Omega (\sigma ). \end{aligned}$$

We use Theorem 1.7 to draw a consequence for the level spacing distribution of the Robin eigenvalues on a rectangle: Let $x_0\le x_1\le x_2\le \ldots$ be a sequence of levels, and $\delta _n=x_{n+1}-x_n$ be the nearest neighbour gaps. We assume that $x_N=N+o(N)$ so that the average gap is unity. The level spacing distribution *P*(*s*) of the sequence is then defined as

$$\begin{aligned} \int _0^y P(s)ds = \lim _{N\rightarrow \infty } \frac{1}{N} \#\{n\le N: \delta _n\le y\} \end{aligned}$$

(assuming that the limit exists).

It is well known that the level spacing distribution for the Neumann (or Dirichlet) eigenvalues on the square is a delta-function at the origin, due to large arithmetic multiplicities in the spectrum. Once we put a Robin boundary condition, we can show [31] that the multiplicities disappear for $\sigma >0$ sufficiently small, except for systematic doubling due to symmetry. Nonetheless, even after desymmetrizing (removing the systematic multiplicities) we show that the level spacing does not change:

#### Theorem 1.8

The level spacing distribution for the desymmetrized Robin spectrum on the square is a delta-function at the origin.

### The disk

As we will explain, upper bounds for the gaps $d_n$ can be obtained from upper bounds for the remainder term in Weyl’s law for the Robin/Neumann problem. While this method will usually fall short of Theorem 1.4, for the disk it gives a better bound. In that case, Kuznetsov and Fedosov [16] (see also Colin de Verdiére [8]) gave an improved remainder term in Weyl’s law for Dirichlet boundary conditions, by relating the problem to counting (shifted) lattice points in a certain cusped domain. With some work, the argument can also be adapted to the Robin case (see Sect. 10.2 and “Appendix A”), which recovers Theorem 1.4 in this special case. The remainder term for the lattice count was improved by Guo, Wang and Wang [13], from which we obtain:

#### Theorem 1.9

For the unit disk, for any fixed $\sigma >0$, we have

$$\begin{aligned} d_n(\sigma ) =O(n^{1/3-\delta }), \quad \delta =1/990. \end{aligned}$$

## Numerics

We present some numerical experiments on the fluctuation of the RN gaps. In all cases, we took the Robin constant to be $\sigma =1$. Displayed are the run sequence plots of the RN gaps. The solid (green) curve is the cumulative mean. The solid (red) horizontal line is the limiting mean value $2{\text {length}}(\partial \Omega )/{\text {area}}(\Omega )$ obtained in Theorem 1.1.

**Fig. 1:**
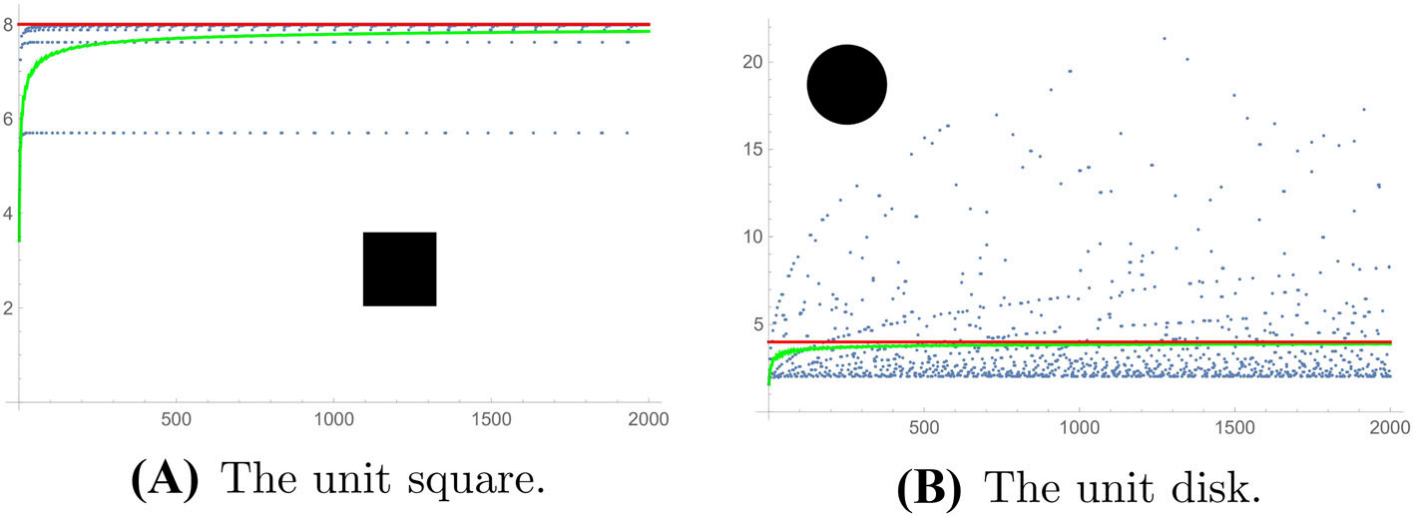
The first 2000 RN gaps for the unit square (**A**) and for the unit disk (**B**)

In Fig. 1 we present numerics for two domains where the Neumann and Dirichlet problems are solvable, by means of separation of variables, the square and the disk. These were generated using Mathematica [35]. For the square, we are reduced to finding Robin eigenvalues on an interval as (numerical) solutions to a secular equation, see Sect. 8, and have used Mathematica to find these.

The disk admits separation of variables, and as is well known the Dirichlet eigenvalues on the unit disk are the squares of the positive zeros of the Bessel functions $J_n(x)$. The positive Neumann eigenvalues are squares of the positive zeros of the derivatives $J_n'(x)$, and the Robin eigenvalues are the squares of the positive zeros of $xJ_n'( x) + \sigma J_n(x)$. We generated these using Mathematica, see Fig. 1B.

**Fig. 2:**
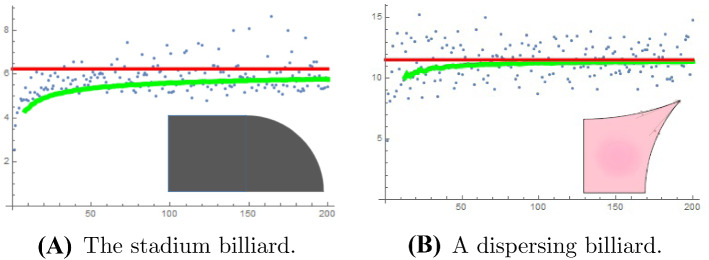
The first 200 RN gaps for the ergodic quarter-stadium billiard (**A**), a quarter of the shape formed by gluing two half-disks to a square of sidelength 2, and for the uniformly hyperbolic billiard consisting of a quarter of the shape formed by the intersection of the exteriors of four disks (**B**)

For the remaining cases we used the finite elements package FreeFem [11, 20]. In Fig. 2 we display two ergodic examples, the quarter-stadium billiard and a uniformly hyperbolic, Sinai-type dispersing billiard which was investigated numerically by Barnett [2].

**Fig. 3:**
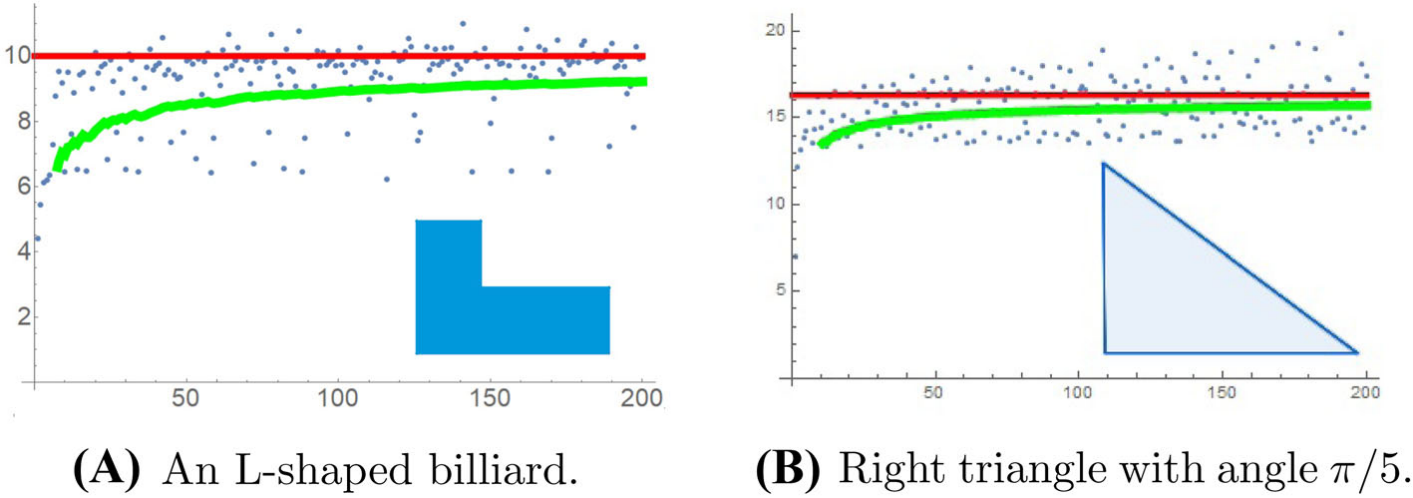
The first 200 RN gaps for two examples of rational polygons: An L-shaped billiard (**A**) made of 4 squares of sidelength 1/2, and a right triangle with an angle $\pi /5$ and a long side of length unity (**B**)

It is also of interest to understand rational polygons, that is simple plane polygons all of whose vertex angles are rational multiples of $\pi$ (Fig. 3), when we expect an analogue of Theorem 1.6 to hold, compare [23].

**Fig. 4:**
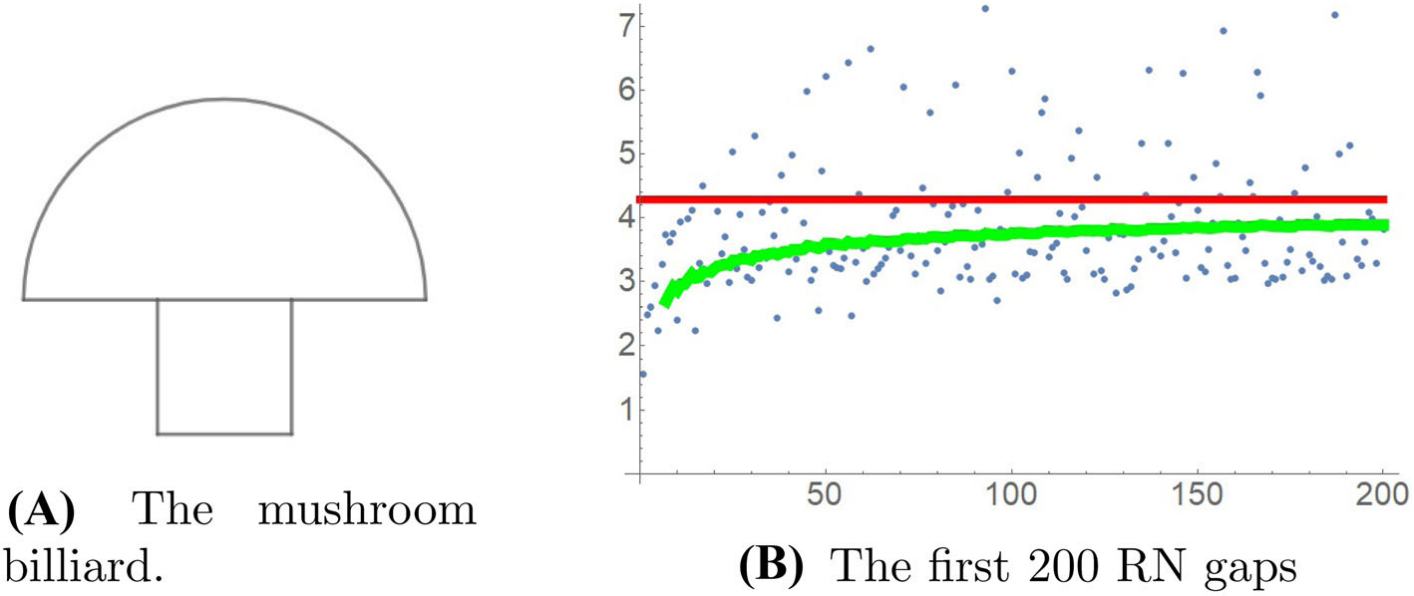
The first 200 RN gaps for the mushroom billiard, with a half-disk of diameter 3 on top of a unit square, which has mixed (chaotic and regular) billiard dynamics

The case of dynamics with a mixed phase space, such as the mushroom billiard investigated by Bunimovich [6] (see also the survey [26]) also deserves study, see Fig. 4.

## Generalities About the RN Gaps

### Robin–Neumann bracketing and positivity of the RN gaps

We recall the min-max characterization of the Robin eigenvalues

$$\begin{aligned} \lambda _n^\sigma = \inf _{\begin{array}{c} M{\subset } H^1(\Omega ) \\ \dim M=n \end{array}} \sup _{0\ne u\in M} \frac{\int _\Omega |\nabla u|^2 dx +\int _{\partial \Omega } \sigma u^2 ds}{\int _\Omega u^2 dx} \end{aligned}$$

where $H^1(\Omega )$ is the Sobolev space. This shows that $\lambda _n^\sigma \ge \lambda _n^0$ if $\sigma >0$. Likewise, there is a min-max characterization of the Dirichlet eigenvalues with $H^1(\Omega )$ replaced by the subspace $H^1_0(\Omega )$, the closure of functions vanishing near the boundary:

$$\begin{aligned} \lambda _n^\infty = \inf _{\begin{array}{c} M{\subset } H^1_0(\Omega ) \\ \dim M=n \end{array}} \sup _{0\ne u\in M} \frac{\int _\Omega |\nabla u|^2 dx }{\int _\Omega u^2 dx} . \end{aligned}$$

This shows that $\lambda _n^\sigma \le \lambda _n^\infty$.

In fact, we have strict inequality,

$$\begin{aligned} \lambda _n^0<\lambda _n^\sigma <\lambda _n^\infty . \end{aligned}$$

This is proved (in greater generality) in [29] using a unique continuation principle.

### A variational formula for the gaps

#### Lemma 3.1

Let $\Omega {\subset } {\mathbb {R}}^d$ be a bounded Lipschitz domain. Then

3.1 $$\begin{aligned} d_n(\sigma ):=\lambda _n^\sigma -\lambda _n^0 = \int _0^\sigma \left( \int _{\partial \Omega } |u_{n,\tau }|^2 ds \right) d\tau \end{aligned}$$

where $u_{n,\tau }$ is any $L^2(\Omega )$-normalized eigenfunction associated with $\lambda _n^\tau$.

#### Proof

According to [1, Lemma 2.11] (who attribute it as folklore), for any bounded Lipschitz domain $\Omega {\subset } {\mathbb {R}}^d$, and $n\ge 1$, the function $\sigma \rightarrow \lambda _n^\sigma$ is strictly increasing for $\sigma \in [0,\infty )$, is differentiable almost everywhere in $(0,\infty )$, is piecewise analytic, and the non-smooth points are locally finite (i.e. finite in each bounded interval). It is absolutely continuous, and in particular its derivative $d\lambda _n^\sigma /d\sigma$ (which exists almost everywhere) is locally integrable, and for any $0\le \alpha <\beta$,

$$\begin{aligned} \lambda _n^\beta - \lambda _n^\alpha = \int _\alpha ^\beta \frac{d\lambda _n^\sigma }{d\sigma }d\sigma . \end{aligned}$$

Moreover, there is a variational formula valid at any point where the derivative exists:

3.2 $$\begin{aligned} \frac{d \lambda _{n}^\sigma }{d\sigma } = \int _{\partial \Omega } |u_{n,\sigma }|^2 ds \end{aligned}$$

where $u_{n,\sigma }$ is any normalized eigenfunction associated with $\lambda _n^\sigma$. Therefore

$$\begin{aligned} d_n(\sigma )=\lambda _n^\sigma -\lambda _n^0 = \int _0^\sigma \frac{d \lambda _{n}^\tau }{d\tau } d\tau = \int _0^\sigma \left( \int _{\partial \Omega } |u_{n,\tau }|^2 ds \right) d\tau . \end{aligned}$$

We can ignore the finitely many points $\tau$ where (3.2) fails, as the derivative is integrable. $\square$

### A general upper bound: Proof of Theorem 1.4

As a corollary, we can show that for the case of smooth boundary, we have an upper bound^1^

$$\begin{aligned} d_n(\sigma )\ll _{\Omega ,\sigma } (\lambda _n^\infty )^{1/3} . \end{aligned}$$

Indeed, for the case of smooth boundary, [3, Proposition 2.4]^2^ give an upper bound on the boundary integrals of eigenfunctions

$$\begin{aligned} \int _{\partial \Omega } u_{n,\sigma }^2 ds\ll _{\Omega } (\lambda _n^\sigma )^{1/3} \le (\lambda _n^\infty )^{1/3}, \end{aligned}$$

uniformly in $$\sigma \ge 0$$.

As a consequence of the variational formula (3.1), we deduce

$$\begin{aligned} d_n(\sigma )\ll _{\Omega } (\lambda _n^\infty )^{1/3} \cdot \sigma \end{aligned}$$

and in particular for planar domains, using Weyl’s law, we obtain for $n\ge 1$

$$\begin{aligned} d_n(\sigma )\ll _{\Omega } n^{1/3}\cdot \sigma . \end{aligned}$$

## The Mean Value

In this section we give a proof of Theorem 1.1, that

$$\begin{aligned} \lim _{N\rightarrow \infty } \frac{1}{N}\sum _{n\le N} d_n(\sigma ) = \frac{2{\text {length}}(\partial \Omega )}{{\text {area}}(\Omega )} \sigma . \end{aligned}$$

Denote

$$\begin{aligned} W_N(\sigma ):= \frac{1}{N}\sum _{n\le N} \int _{\partial \Omega } u_{n,\sigma }^2 ds . \end{aligned}$$

Using Lemma 3.1 gives

$$\begin{aligned} \frac{1}{N} \sum _{n=1}^N d_n(\sigma ) = \int _0^\sigma \left( \frac{1}{N}\sum _{n\le N} \int _{\partial \Omega } u_{n,\tau }^2 ds\right) d\tau = \int _0^\sigma W_N(\tau ) d\tau . \end{aligned}$$

The local Weyl law [14] (valid for any piecewise smooth $\Omega$) shows that for any fixed $\sigma$,

$$\begin{aligned} \lim _{N\rightarrow \infty } W_N(\sigma ) = \frac{2{\text {length}}(\partial \Omega )}{{\text {area}}(\Omega )} \end{aligned}$$

so if we know that $W_N(\tau )\le C$ is uniformly bounded for all $\tau \le \sigma$, then by the Dominated Convergence Theorem we deduce that

$$\begin{aligned} \lim _{N\rightarrow \infty } \frac{1}{N} \sum _{n=1}^N d_n(\sigma )= & {} \int _0^\sigma \lim _{N\rightarrow \infty } W_N(\tau ) d\tau \\= & {} \int _0^\sigma \frac{2{\text {length}}(\partial \Omega )}{{\text {area}}(\Omega )} d\tau = \frac{2{\text {length}}(\partial \Omega )}{{\text {area}}(\Omega )} \cdot \sigma \end{aligned}$$

as claimed.

It remains to prove a uniform upper bound for $W_N(\sigma )$.

### Lemma 4.1

There is a constant $C=C(\Omega )$ so that for all $\sigma >0$ and all $N\ge 1$,

$$\begin{aligned} \frac{1}{N}\sum _{n\le N} \int _{\partial \Omega } u_{n,\sigma }^2 ds \le C . \end{aligned}$$

### Proof

What we use is an upper bound on the heat kernel *on the boundary*. Let $K_\sigma (x,y;t)$ be the heat kernel for the Robin problem. Then [14, Lemma 12.1],

4.1 $$\begin{aligned} K_\sigma (x,y;t)\le C t^{-\dim \Omega /2}\exp (-\delta | x-y|^2/t) \end{aligned}$$

where $C,\delta >0$ depend only on the domain $\Omega$. Moreover, on the regular part of the boundary,

$$\begin{aligned} K_\sigma (x,y;t) = \sum _{n\ge 0}e^{-t\lambda _n^\sigma } u_{n,\sigma }(x) u_{n,\sigma }(y) . \end{aligned}$$

We have for $n\le N$ that $\lambda _n^\sigma \le \lambda _N^\sigma \le \lambda _N^\infty$, so for $\Lambda =\lambda _N^\infty$,

$$\begin{aligned} \sum _{n\le N} \int _{\partial \Omega } u_{n,\sigma }^2 ds \le e \sum _{n\le N} e^{-\lambda _n^\sigma /\Lambda }\int _{\partial \Omega } u_{n,\sigma }^2 ds \le e \int _{\partial \Omega } K_\sigma \left( x,x;\frac{1}{\Lambda }\right) ds . \end{aligned}$$

By (4.1),

$$\begin{aligned} \int _{\partial \Omega } K_\sigma \left( x,x;\frac{1}{\Lambda }\right) ds \ll _\Omega \Lambda ^{\dim \Omega /2} . \end{aligned}$$

Thus we find a uniform upper bound

$$\begin{aligned} \sum _{\lambda _n^\sigma \le \Lambda } \int _{\partial \Omega } u_{n,\sigma }^2 ds \ll _\Omega \Lambda ^{\dim \Omega /2} \approx N \end{aligned}$$

on using Weyl’s law, that is for all $\sigma >0$

$$\begin{aligned} \frac{1}{N} \sum _{n\le N} \int _{\partial \Omega } u_{n,\sigma }^2 ds \le C(\Omega ) . \end{aligned}$$

$$\square $$

We note that the mean value result is valid in any dimension $d\ge 2$ for piecewise smooth domains $\Omega {\subset }{\mathbb {R}}^d$ as in [14], in the form

$$\begin{aligned} \lim _{N\rightarrow \infty } \frac{1}{N}\sum _{n\le N} d_n(\sigma ) = \frac{2{\text {vol}}_{d-1}(\partial \Omega )}{{\text {vol}}_d (\Omega )} \sigma . \end{aligned}$$

Indeed [14] prove the local Weyl law in that context, and Lemma 4.1 is also valid in any dimension.

## A Uniform Lower Bound for the Gaps

To obtain the lower bound of Theorem 1.3 for the gaps, we use the variational formula (3.1) to relate the derivative $d\lambda _n^\sigma /d\sigma$ to the boundary integrals $\int _{\partial \Omega }u_{n,\sigma }^2 ds$, where $u_{n,\sigma }$ is any eigenfunction with eigenvalue $\lambda _n^\sigma$, and for that will require a lower bound on these boundary integrals.

### A lower bound for the boundary integral

The goal here is to prove a uniform lower bound for the boundary data of Robin eigenfunctions on a star-shaped, smooth planar domain $\Omega$.

#### Theorem 5.1

Let $\Omega {\subset } {\mathbb {R}}^2$ be a star-shaped bounded planar domain with smooth boundary. Let *f* be an $L^2(\Omega )$ normalized Robin eigenfunction associated with the *n*-th eigenvalue $\lambda _n^\sigma$. Then there are constants $C>0$, $A,B\ge 0$ depending on $\Omega$ so that for all $n\ge 1$,

5.1 $$\begin{aligned} \int _{\partial \Omega } f^2 ds \ge \frac{1}{A\sigma ^2 +B\sigma +C}>0. \end{aligned}$$

For $\sigma =0$ (Neumann problem), this is related to the $L^2$ restriction bound of Barnett–Hassell–Tacy [3, Proposition 6.1].

### The Neumann case $\sigma =0$

We first show the corresponding statement for Neumann eigenfunctions (which are Robin case with $\sigma =0$), which is much simpler. Let *f* be a Neumann eigenfunction, that is $(\Delta +\lambda )f=0$ in $\Omega$, $\frac{\partial f}{\partial n}=0$ in $\partial \Omega$. We may assume that $\lambda >0$, the result being obvious for $\lambda =0$ when *f* is a constant function. After translation, we may assume that the domain is star-shaped with respect to the origin.

We start with a Rellich identity ([27, Eq 2]): Assume that $\Omega {\subset } {\mathbb {R}}^d$ is a Lipschitz domain. Let $L=\Delta +\lambda$, and $A=\sum _{j=1}^d x_j\frac{\partial }{\partial x_j}$. For every function *f* on $\Omega$

5.2 $$\begin{aligned}&\int _\Omega (Lf)(Af) dx = \int _{\partial \Omega } \frac{\partial f}{\partial n}Af -\frac{1}{2} \int _{\partial \Omega }||\nabla f||^2 \left( \sum _{j=1}^d x_j\frac{\partial x_j}{\partial n} \right) \nonumber \\&\quad + \frac{\lambda }{2} \int _{\partial \Omega } f^2 \left( \sum _{j=1}^d x_j\frac{\partial x_j}{\partial n} \right) -\frac{d}{2}\lambda \int _{\Omega } f^2 dx + \left( \frac{d}{2}-1\right) \int _\Omega ||\nabla f||^2 dx . \end{aligned}$$

Using (5.2) in dimension $d=2$ for a normalized eigenfunction, so that $Lf=0$ and $\int _\Omega f^2 =1$, and recalling that for Neumann eigenfunctions $\frac{\partial f}{\partial n}=0$ on $\partial \Omega$, gives

$$\begin{aligned} 0=-\frac{1}{2} \int _{\partial \Omega }||\nabla f||^2 \left( x\frac{\partial x}{\partial n} + y\frac{\partial y}{\partial n} \right) + \frac{\lambda }{2} \int _{\partial \Omega } f^2 \left( x\frac{\partial x}{\partial n} + y\frac{\partial y}{\partial n} \right) -\lambda \end{aligned}$$

or

$$\begin{aligned} \int _{\partial \Omega } \left( f^2-\frac{1}{\lambda }||\nabla f||^2 \right) \left( x\frac{\partial x}{\partial n} + y\frac{\partial y}{\partial n} \right) ds = 2 . \end{aligned}$$

The term $x\frac{\partial x}{\partial n} + y\frac{\partial y}{\partial n}$ is the inner product $n(\mathbf {x}) \cdot \mathbf {x}$ between the outward unit normal $n(\mathbf {x}) = (\frac{\partial x}{\partial n} , \frac{\partial y}{\partial n})$ at the point $\mathbf {x}\in \partial \Omega$ and the radius vector $\mathbf {x}=(x,y)$ joining $\mathbf {x}$ and the origin. Since the domain is star-shaped w.r.t. the origin, we have on the boundary $\partial \Omega$

$$\begin{aligned} x\frac{\partial x}{\partial n} + y\frac{\partial y}{\partial n} = n(\mathbf {x}) \cdot \mathbf {x} \ge 0 \end{aligned}$$

so that we can drop^3^ the term with $||\nabla f||^2$ and get an inequality

$$\begin{aligned} \int _{\partial \Omega } (n(\mathbf {x}) \cdot \mathbf {x} ) f^2 ds \ge 2. \end{aligned}$$

Replacing $(n(\mathbf {x}) \cdot \mathbf {x} ) \le 2C_\Omega$ on $\partial \Omega$ gives Theorem 5.1 for $\sigma =0$:

$$\begin{aligned} \int _{\partial \Omega } f^2 \ge \frac{1}{C_\Omega } . \end{aligned}$$

### The Robin case

Using the Rellich identity (5.2) in dimension $d=2$ for a normalized eigenfunction, so that $Lf=0$ and $\int _\Omega f^2 =1$, gives

$$\begin{aligned} 0= \int _{\partial \Omega } \frac{\partial f}{\partial n}Af -\frac{1}{2} \int _{\partial \Omega }||\nabla f||^2 (n(\mathbf {x}) \cdot \mathbf {x} ) + \frac{\lambda }{2} \int _{\partial \Omega } f^2 (n(\mathbf {x}) \cdot \mathbf {x} ) -\lambda . \end{aligned}$$

Now $n(\mathbf {x}) \cdot \mathbf {x}\ge 0$ on the boundary $\partial \Omega$ since $\Omega$ is star-shaped with respect to the origin, and $\lambda >0$, so we may drop the term with $||\nabla f||^2$ and get an inequality

$$\begin{aligned} \int _{\partial \Omega } f^2(\mathbf {x}) (n(\mathbf {x}) \cdot \mathbf {x}) ds + \frac{2}{\lambda }\int _{\partial \Omega } \frac{\partial f}{\partial n}Af \ge 2 . \end{aligned}$$

Due to the boundary condition, we may replace the normal derivative $\frac{\partial f}{\partial n}$ by $-\sigma f$, and obtain, after using $0 \le n(\mathbf {x}) \cdot \mathbf {x} \le 2C=2C_\Omega$ (we may take 2*C* to be the diameter of $\Omega$), that

5.3 $$\begin{aligned} C \int _{\partial \Omega } f^2 - \frac{ \sigma }{\lambda }\int _{\partial \Omega } f (Af )\ge 1 . \end{aligned}$$

To proceed further, we need:

#### Lemma 5.2

Assume that $\partial \Omega$ is smooth. There are numbers $P,Q\ge 0$, not both zero, depending only on $\partial \Omega$, so that for any normalized $\sigma$-Robin eigenfunction *f*,

5.4 $$\begin{aligned} \left| \int _{\partial \Omega } f (Af) ds \right| \le (P+\sigma Q) \int _{\partial \Omega } f^2ds . \end{aligned}$$

#### Proof

Decompose the vector field $A=x\frac{\partial }{\partial x} + y\frac{\partial }{\partial y}$ into its normal and tangential components along the boundary:

$$\begin{aligned} A =p \frac{\partial }{\partial n} + q \frac{\partial }{\partial \tau } \end{aligned}$$

where *p*, *q* are functions on the boundary $\Omega$. For example, for the circle $x^2+y^2=\rho ^2$, we have $A=\rho \frac{\partial }{\partial n}$ and the normal derivative is just the radial derivative $\frac{\partial }{\partial n}= \frac{\partial }{\partial r}$, so that $p \equiv \rho$, and $q \equiv 0$.

Then using the Robin condition $\frac{\partial f}{\partial n} =-\sigma f$ on $\partial \Omega$ gives

$$\begin{aligned} \int _{\partial \Omega } f (Af) ds = \int _{\partial \Omega } f \left( p \frac{\partial f}{\partial n} +q \frac{\partial f}{\partial \tau }\right) ds = -\sigma \int _{\partial \Omega }p f^2 ds+ \int _{\partial \Omega } q f \frac{\partial f}{\partial \tau } ds . \end{aligned}$$

Setting $P:=\max _{\partial \Omega }|p|$, we have

$$\begin{aligned} \left| -\sigma \int _{\partial \Omega }p f^2 ds\right| \le \sigma P \int _{\partial \Omega } f^2ds \end{aligned}$$

so it remains to bound $\left| \int _{\partial \Omega } q f \frac{\partial f}{\partial \tau } ds\right|$.

Let $\gamma :[0,L]\rightarrow \partial \Omega$ be an arclength parameterization with $\gamma (0) = \gamma (L)$. Then note that the tangential derivative of *f* at $x_0 = \gamma (s_0)$ is

$$\begin{aligned} \frac{\partial f}{\partial \tau }(x_0) = \frac{d}{ds}f(\gamma (s))\Big |_{s=s_0} \end{aligned}$$

and hence

$$\begin{aligned} f \frac{\partial f}{\partial \tau } = \frac{1}{2} \frac{\partial (f^2) }{\partial \tau }=\frac{1}{2} \frac{d }{ds} \left\{ f(\gamma (s))^2\right\} \end{aligned}$$

so that abbreviating $q(s) = q(\gamma (s))$ and integrating by parts

$$\begin{aligned} \begin{aligned} \int _{\partial \Omega } q f \frac{\partial f}{\partial \tau } ds&= \frac{1}{2} \int _0^L q(s) \frac{d }{ds} \left\{ f(\gamma (s))^2\right\} ds \\&= \frac{1}{2} q(s)f(\gamma (s))^2\Big |_0^L - \frac{1}{2} \int _0^L q'(s) f(\gamma (s))^2 ds . \end{aligned} \end{aligned}$$

Because the curve is closed: $\gamma (L)=\gamma (0)$, the boundary terms cancel out:

$$\begin{aligned} q(s)f(\gamma (s))^2\Big |_0^L = q(\gamma (L)) f(\gamma (L))^2- q(\gamma (0)) f(\gamma (0))^2 =0 \end{aligned}$$

and so

$$\begin{aligned} \left| \int _{\partial \Omega } q f \frac{\partial f}{\partial \tau } ds\right| = \left| \frac{1}{2} \int _0^L q'(s) f(\gamma (s))^2 ds\right| \le Q \int _{\partial \Omega } f^2 ds \end{aligned}$$

where $Q=\max _{\partial \Omega }|\frac{dq}{d\tau }|$. Altogether we found that

$$\begin{aligned} \left| \int _{\partial \Omega } f (Af) ds \right| \le (\sigma P+Q) \int _{\partial \Omega } f^2 ds . \end{aligned}$$

$$\square $$

We may now conclude the proof of Theorem 5.1 for $\sigma >0$: Take $f=u_{n,\sigma }$ the *n*-th eigenfunction, with $n\ge 1$. Inserting (5.4) into (5.3) we find

$$\begin{aligned} 1\le C \int _{\partial \Omega } f^2 - \frac{ \sigma }{\lambda }\int _{\partial \Omega } f (Af ) \le \left( C+\frac{ \sigma (P+Q\sigma )}{\lambda _n^\sigma } \right) \int _{\partial \Omega } f^2 ds . \end{aligned}$$

Hence we find, on replacing $\lambda _n^{\sigma }\ge \lambda _1^{\sigma } \ge \lambda _1^0>0$, that

$$\begin{aligned} \int _{\partial \Omega } f^2 ds \ge \frac{1}{C+ \sigma (P+Q\sigma )/ \lambda _1^0}>0 \end{aligned}$$

which is of the desired form. $\quad \square$

### Proof of Theorem 1.3

We use the variational formula (3.1) for $n\ge 1$ with the lower bound (5.1) of Theorem 5.1

$$\begin{aligned} d_n(\sigma ) = \int _0^\sigma \left( \oint _{\partial \Omega } u_{n,\tau }^2 ds \right) d\tau \ge \int _0^\sigma \frac{d\tau }{A\tau ^2+B\tau +C } =:c_1(\Omega ,\sigma ) >0. \end{aligned}$$

For $n=0$, we just use positivity of the RN gap $d_0(\sigma )>0$, and finally deduce that for all $n\ge 0$, and $\sigma >0$,

$$\begin{aligned} d_n(\sigma )\ge c(\Omega ,\sigma ):=\min \left( c_1(\Omega ,\sigma ), d_0(\sigma ) \right) >0. \end{aligned}$$

$$\square $$

## Ergodic Billiards

In this section we give a proof of Theorem 1.6. By Chebyshev’s inequality, it suffices to show:

### Proposition 6.1

Let $\Omega {\subset } {\mathbb {R}}^2$ be a bounded, piecewise smooth domain. Assume that the billiard map for $\Omega$ is ergodic. Then for every $\sigma >0$,

6.1 $$\begin{aligned} \lim _{N\rightarrow \infty } \frac{1}{N}\sum _{n\le N} \left| d_n(\sigma )-\frac{2{\text {length}}(\partial \Omega )}{{\text {area}}(\Omega )} \cdot \sigma \right| = 0 . \end{aligned}$$

### Proof

We again use the variational formula (3.1)

$$\begin{aligned} d_n(\sigma ) = \int _0^\sigma \left( \int _{\partial \Omega } u_{n,\tau }^2 ds \right) d\tau . \end{aligned}$$

We have

$$\begin{aligned} \begin{aligned} \left| d_n(\sigma )- \frac{2{\text {length}}(\partial \Omega )}{{\text {area}}(\Omega )}\sigma \right|&= \left| \int _0^\sigma \Big ( \int _{\partial \Omega } u_{n,\tau }^2 ds \Big ) d\tau - \frac{2{\text {length}}(\partial \Omega )}{{\text {area}}(\Omega )} \sigma \right| \\&= \left| \int _0^\sigma \Big (\int _{\partial \Omega } u_{n,\tau }^2 ds - \frac{2{\text {length}}(\partial \Omega )}{{\text {area}}(\Omega )} \Big ) d\tau \right| \\&\le \int _0^\sigma \left| \int _{\partial \Omega } u_{n,\tau }^2 ds - \frac{2{\text {length}}(\partial \Omega )}{{\text {area}}(\Omega )} \right| d\tau . \end{aligned} \end{aligned}$$

Therefore

$$\begin{aligned} \begin{aligned} \frac{1}{N}\sum _{n\le N} \left| d_n(\sigma )- \frac{2{\text {length}}(\partial \Omega )}{{\text {area}}(\Omega )}\sigma \right|&\le \int _0^\sigma \frac{1}{N}\sum _{n\le N} \left| \int _{\partial \Omega } u_{n,\tau }^2 ds - \frac{2{\text {length}}(\partial \Omega )}{{\text {area}}(\Omega )} \right| d\tau \\&=: \int _0^\sigma S_N(\tau ) d\tau \end{aligned} \end{aligned}$$

where

$$\begin{aligned} S_N(\tau ):= \frac{1}{N}\sum _{n\le N} \left| \int _{\partial \Omega } u_{n,\tau }^2 ds - \frac{2{\text {length}}(\partial \Omega )}{{\text {area}}(\Omega )} \right| . \end{aligned}$$

Hassell and Zelditch [14, eq 7.1] (see also Burq [7]) show that if the billiard map is ergodic then for each $\sigma \ge 0$,

6.2 $$\begin{aligned} \lim _{N\rightarrow \infty } \frac{1}{N}\sum _{n\le N} \left| \int _{\partial \Omega } u_{n,\sigma }^2 ds-\frac{2{\text {length}}(\partial \Omega )}{{\text {area}}(\Omega )} \right| ^2 = 0 . \end{aligned}$$

Therefore, by Cauchy–Schwarz, $S_N(\tau )$ tends to zero for all $\tau \ge 0$, by (6.2); by Lemma 4.1 we know that $S_N(\tau )\le C$ is uniformly bounded for all $\tau \le \sigma$, so that by the Dominated Convergence Theorem we deduce that the limit of the integrals tends to zero, hence that

$$\begin{aligned} \lim _{N\rightarrow \infty } \frac{1}{N}\sum _{n\le N} \left| d_n(\sigma )- \frac{2{\text {length}}(\partial \Omega )}{{\text {area}}(\Omega )}\sigma \right| = 0. \end{aligned}$$

$$\square $$

We note that Theorem 1.6 is valid in any dimension $d\ge 2$ for piecewise smooth domains $\Omega {\subset }{\mathbb {R}}^d$ with ergodic billiard map as in [14], with the mean value interpreted as $\frac{2{\text {vol}}_{d-1}(\partial \Omega )}{{\text {vol}}_d (\Omega )} \sigma$.

## Variable Robin Function

In this section, we indicate extensions of our general results to the case of variable boundary conditions.

### Variable boundary conditions

The general Robin boundary condition is obtained by taking a function on the boundary $\sigma : \partial \Omega \rightarrow {\mathbb {R}}$ which we assume is always non-negative: $\sigma (x)\ge 0$ for all $x\in \partial \Omega$. Thus we look for solutions of

$$\begin{aligned}&\Delta u +\lambda u=0 \;\mathrm{on} \;\Omega , \\&\frac{\partial u}{\partial n}(x) +\sigma (x) u(x) = 0, \quad x\in \partial \Omega \end{aligned}$$

which is interpreted in weak form as saying that

$$\begin{aligned} \int _\Omega \nabla u_{n } \cdot \nabla v +\oint _{\partial \Omega } \sigma u_{n} v = \lambda _n \int _\Omega u_{n } v \end{aligned}$$

for all $v\in H^1(\Omega )$. We will assume that $\sigma$ is continuous. Then we obtain positive Robin eigenvalues

$$\begin{aligned} 0<\lambda _0^\sigma \le \lambda _1^\sigma \le \ldots \end{aligned}$$

except that in the Neumann case $\sigma \equiv 0$ we also have zero as an eigenvalue.

Robin to Neumann bracketing is still valid here, in the following form: if $\sigma _1,\sigma _2\in C(\partial \Omega )$ are two continuous functions with $0\le \sigma _1\le \sigma _2$ and such that there is some point $x_0\in \partial \Omega$ such that there is strict inequality $\sigma _1(x_0)<\sigma _2(x_0)$ (by continuity this therefore holds on a neighborhood of $x_0$), then we have a strict inequality [29]

7.1 $$\begin{aligned} \lambda _n^{\sigma _1}<\lambda _n^{\sigma _2}, \quad \forall n\ge 1. \end{aligned}$$

Fix such a Robin function $\sigma \in C(\partial \Omega )$, which is positive: $\sigma (x)>0$ for all $x\in \partial \Omega$. We are interested in the Robin–Neumann gaps

$$\begin{aligned} d_n(\sigma ):=\lambda _n^\sigma - \lambda _n^0 \end{aligned}$$

which are positive by (7.1).

### Extension of general results

The lower and upper bounds of Theorems 1.3 and 1.4 remain valid for variable $\sigma$ by an easy reduction to the constant case: Let

$$\begin{aligned} \sigma _{\min }=\min _{x\in \partial \Omega } \sigma (x), \quad \sigma _{\max }=\max _{x\in \partial \Omega } \sigma (x) \end{aligned}$$

so that $0<\sigma _{\min }\le \sigma _{\max }$ (with equality only if $\sigma$ is constant). Using (7.1) gives

$$\begin{aligned} \lambda _n^0<\lambda _n^{\sigma _{\min }}\le \lambda _n^{\sigma }\le \lambda _n^{\sigma _{\max }} \end{aligned}$$

so that

$$\begin{aligned} d_n(\sigma _{\min })\le d_n(\sigma )\le d_n(\sigma _{\max }). \end{aligned}$$

For instance, the universal lower bound for star-shaped domains (Theorem 1.3) follows because $d_n(\sigma )\ge d_n(\sigma _{\min }) \ge C(\sigma _{\min })>0$, etcetera.

The existence of mean values (Theorem 1.1) and the almost sure convergence of the gaps to the mean value in the ergodic case (Theorem 1.6) require an adjustment of the variational formula (Lemma 3.1) which is provided in Sect. 7.3. Once that is in place, the result is

7.2 $$\begin{aligned} \lim _{N\rightarrow \infty } \frac{1}{N} \sum _{n=1}^N d_n(\sigma ) = \frac{2\oint _{\partial \Omega }\sigma (s)ds}{{\text {area}}(\Omega )} . \end{aligned}$$

Given the mean value formula (7.2), Theorem 1.6 (almost sure convergence of the RN gaps to the mean in the ergodic case) also follows.

### A variational formula

Let $\Omega {\subset } {\mathbb {R}}^d$ be a bounded Lipschitz domain. Fix a continuous, positive Robin function $\sigma :\partial \Omega \rightarrow {\mathbb {R}}_{>0}$. We consider a one-parameter deformation of the boundary value problem $\Delta u+\lambda u=0$,

7.3 $$\begin{aligned} \frac{\partial u}{\partial n}(x) +\alpha \sigma (x) u(x) = 0, \quad x\in \partial \Omega \end{aligned}$$

with a real parameter $\alpha \ge 0$. Denote the corresponding eigenvalues by

$$\begin{aligned} \lambda _1(\alpha )\le \lambda _2(\alpha )\le \cdots \le \lambda _n(\alpha )\le \cdots \end{aligned}$$

By Robin–Neumann bracketing, if $0\le \alpha _1<\alpha _2$ then

$$\begin{aligned} \lambda _n(\alpha _1)<\lambda _n(\alpha _2), \quad \forall n\ge 1 . \end{aligned}$$

The previous RN gaps $d_n(\sigma )$ are precisely $\lambda _n(1)-\lambda _n(0)$. The variational formula for the RN gaps is:

#### Lemma 7.1

Let $\Omega {\subset } {\mathbb {R}}^d$ be a bounded Lipschitz domain. Then

$$\begin{aligned} d_n(\sigma ) = \int _0^1 \left( \int _{\partial \Omega } |u_{n,\alpha }|^2 ds \right) d\alpha \end{aligned}$$

where $u_{n,\alpha }$ is any $L^2(\Omega )$-normalized eigenfunction associated with $\lambda _n(\alpha )$.

The proof is identical to that of Lemma 3.1, except that we need a reformulation of [1, Lemma 2.11] to this context^4^:

#### Lemma 7.2

Let $\Omega {\subset } {\mathbb {R}}^d$ be a bounded Lipschitz domain and $\sigma$ a continuous function on the boundary $\partial \Omega$ which is positive: $\sigma (x)>0$ for all $x\in \partial \Omega$. For $\alpha \ge 0$, let $\lambda _n(\alpha )$ be the eigenvalues of the Robin eigenvalue problem (7.3)

Then for $n \ge 1$, $\lambda _n(\alpha )$ is an absolutely continuous and strictly increasing function of $\alpha \in [0,\infty )$, which is differentiable almost everywhere in $(0,\infty )$. Where it exists, its derivative is given by

7.4 $$\begin{aligned} \frac{d}{d\alpha } \lambda _n(\alpha ) = \frac{ \oint _{\partial \Omega } \sigma u_{n,\alpha }^2 }{\int _\Omega u_{n,\alpha }^2} \end{aligned}$$

where $u_{n,\alpha }\in H^1(\Omega )$ is any eigenfunction associated with $\lambda _n(\alpha )$.

#### Proof

The proof is verbatim that of [1, Lemma 2.11] where $\sigma \equiv 1$. As is explained there, each eigenvalue depends locally analytically on $\alpha$, with at most a locally finite set of splitting points. We just repeat the computation of the derivative at any $\alpha$ which is not a splitting point for $\lambda _n(\alpha )$: We use the weak formulation of the boundary condition, as saying that for all $v\in H^1(\Omega )$,

7.5 $$\begin{aligned} \int _\Omega \nabla u_{n,\alpha } \cdot \nabla v +\oint _{\partial \Omega } \alpha \sigma (s) u_{n,\alpha }(s) v(s) ds = \lambda _n(\alpha ) \int _\Omega u_{n,\alpha } v . \end{aligned}$$

In particular, applying (7.5) with $v=u_{n,\beta }$ gives

7.6 $$\begin{aligned} \int _\Omega \nabla u_{n,\alpha } \cdot \nabla u_{n,\beta } +\oint _{\partial \Omega } \alpha \sigma u_{n,\alpha } u_{n,\beta } ds = \lambda _n(\alpha ) \int _\Omega u_{n,\alpha } u_{n,\beta }. \end{aligned}$$

Changing the roles of $\alpha$ and $\beta$ gives

7.7 $$\begin{aligned} \int _\Omega \nabla u_{n,\alpha } \cdot \nabla u_{n,\beta } +\oint _{\partial \Omega } \beta \sigma u_{n,\alpha } u_{n,\beta } ds = \lambda _n(\beta ) \int _\Omega u_{n,\alpha } u_{n,\beta }. \end{aligned}$$

Subtracting (7.6) from (7.7) gives

$$\begin{aligned} \frac{\lambda _n(\beta )-\lambda _n(\alpha )}{\beta -\alpha } = \frac{\oint _{\partial \Omega } \sigma (s) u_{n,\alpha }(s) u_{n,\beta }(s) ds }{ \int _\Omega u_{n,\alpha } u_{n,\beta }} . \end{aligned}$$

Taking the limit $\beta \rightarrow \alpha$ and assuming that $u_{n,\beta }\rightarrow u_{n,\alpha }$ in $H^1(\Omega )$ as $\beta \rightarrow \alpha$, as verified in [1, Lemma 2.11] so that in particular the denominator is eventually nonzero, gives (7.4). $\quad \square$

## Boundedness of RN Gaps for Rectangles

We consider the rectangle $Q_{L}=[0,1]\times [0,L]$, with $L\in (0,1]$ the aspect ratio. We denote by $\lambda _0^\sigma \le \lambda _1^\sigma \le \ldots$ the ordered Robin eigenvalues. We will prove Theorem 1.7, that

$$\begin{aligned} 0<\lambda _n^\sigma -\lambda _n^0 <C_L(\sigma ) . \end{aligned}$$

### The one-dimensional case

Let $\sigma >0$ be the Robin constant. The Robin problem on the unit interval is $-u_n''=k_n^2 u_n$, with the one-dimensional Robin boundary conditions

$$\begin{aligned} -u'(0) +\sigma u(0)=0, \quad u'(1)+\sigma u(1)=0 . \end{aligned}$$

The eigenvalues of the Laplacian on the unit interval are the numbers $-k_n^2$ where the frequencies $k_n=k_n(\sigma )$ are the solutions of the secular equation $(k^2-\sigma ^2)\sin k = 2k\sigma \cos k$, or

**Fig. 5:**
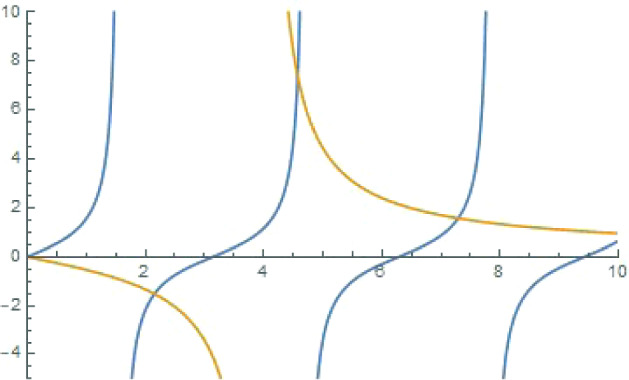
The secular equation (8.1) for $\sigma =4$. Displayed are plots of $\tan k$ versus $\frac{2\sigma k}{k^2-\sigma ^2}$

8.1 $$\begin{aligned} \tan (k) =\frac{2\sigma k}{k^2-\sigma ^2} \end{aligned}$$

(see Fig. 5) and the corresponding eigenfunctions are

$$\begin{aligned} u_n(x)=k_n\cos (k_nx)+\sigma \sin (k_n x). \end{aligned}$$

As a special case^5^ of Dirichlet–Neumann bracketing (1.1), we know that given $\sigma >0$, for each $n\ge 0$ there is a unique solution $k_n=k_n(\sigma )$ of the secular equation (8.1) with

$$\begin{aligned} k_n\in ( n\pi , (n+1)\pi ), \quad n\ge 0. \end{aligned}$$

Note that $$k_n(0) = n\pi $$.

From (8.1), we have as $n\rightarrow \infty$,

$$\begin{aligned} k_n(\sigma )=n\pi +\arctan \left( \frac{2\sigma }{k_n(\sigma )} \frac{1}{1-\frac{\sigma ^2}{k_n(\sigma )^2}} \right) =n\pi +\frac{2\sigma }{k_n(\sigma )} +O\left( k_n(\sigma )^{-3}\right) \end{aligned}$$

so that

8.2 $$\begin{aligned} k_n(\sigma )^2-k_n(0)^2 \sim 4\sigma , \quad n\rightarrow \infty . \end{aligned}$$

We can interpret, for $\Omega$ being the unit interval, $4=2\#\partial \Omega /{\text {length}}\Omega$ so that we find convergence of the RN gaps to their mean value in this case.

From (8.2) we deduce:

#### Lemma 8.1

For every $\sigma >0$, there is some $C(\sigma )>0$ so that

8.3 $$\begin{aligned} k_n(\sigma )^2-k_n(0)^2 \le C(\sigma ), \quad \forall n\ge 0. \end{aligned}$$

### Proof of Theorem 1.7

The frequencies for the interval [0, *L*] are $\frac{1}{L}\cdot k_{m}(\sigma \cdot L)$. Hence the Robin energy levels of $Q_{L}$ are the numbers

8.4 $$\begin{aligned} \Lambda _{n,m}(\sigma )=k_{n}(\sigma )^{2}+\frac{1}{L^{2}}\cdot k_{m}(\sigma \cdot L)^{2}, \quad n,m\ge 0. \end{aligned}$$

We have

$$\begin{aligned} 0\le \Lambda _{n,m}(\sigma )-\Lambda _{n,m}(0) =(k_{n}(\sigma )^{2}-k_{n}(0)^{2}) + \frac{1}{L^{2}}\cdot \left( k_{m}(\sigma \cdot L)^{2}-k_{m}(0)^2\right) . \end{aligned}$$

From the one-dimensional result (8.3), we deduce that

$$\begin{aligned} \Lambda _{n,m}(\sigma )-\Lambda _{n,m}(0) \le C(\sigma ) + \frac{1}{L^2}C(L\sigma ) = C_L(\sigma ). \end{aligned}$$

We now pass from the $\Lambda _{m,n}(\sigma )$ to the ordered eigenvalues $\{\lambda _k^\sigma : k=0,1,\ldots \}$. We know that $\lambda _k^\sigma \ge \lambda _k^0$, and want to show that $\lambda _k^\sigma \le \lambda _k^0+C_L(\sigma )$. For this it suffices to show that the interval $I_k:=[0,\lambda _k^0+C_L(\sigma )]$ contains at least $k+1$ Robin eigenvalues, since then it will contain $\lambda _0^\sigma ,\ldots , \lambda _k^\sigma$ and hence we will find $\lambda _k^\sigma \le \lambda _k^0+C_L(\sigma )$.

The interval $I_k$ contains the interval $[0,\lambda _k^0]$ and so certainly contains the first $k+1$ Neumann eigenvalues $\lambda _0^0,\ldots ,\lambda _k^0$, which are of the form $\Lambda _{m,n}(0)$ with (*m*, *n*) lying in a set ${\mathcal {S}}_k$. Since $\Lambda _{m,n}(\sigma )\le \Lambda _{m,n}(0)+C_L(\sigma )$, the interval $I_k$ must contain the $k+1$ eigenvalues $\{\Lambda _{m,n}(\sigma ) :(m,n)\in {\mathcal {S}}_k\}$, and we are done. $\quad \square$

## Application of Boundedness of the RN Gaps to Level Spacings

In this section, we show that the level spacing distribution of the Robin eigenvalues for the desymmetrized square is a delta function at the origin, as is the case with Neumann or Dirichlet boundary conditions.

Recall the definition of the level spacing distribution: We are given a sequence of levels $x_0\le x_1\le x_2\le \ldots$. We assume that $x_N=cN+o(N)$, as is the case of the eigenvalues of a planar domain. Let $\delta _n=(x_{n+1}-x_n)/c$ be the normalized nearest neighbour gaps. so that the average gap is unity. The level spacing distribution *P*(*s*) of the sequence is then defined as

$$\begin{aligned} \int _0^y P(s)ds = \lim _{N\rightarrow \infty } \frac{1}{N} \#\{n\le N: \delta _n\le y\} \end{aligned}$$

(assuming that the limit exists).

Recall that the Robin spectrum has systematic double multiplicities $\Lambda _{m,n}(\sigma ) = \Lambda _{n,m}(\sigma )$ (see (8.4) with $L=1$), which forces half the gaps to vanish for a trivial reason. To avoid this issue, one takes only the levels $\Lambda _{m,n}(\sigma )$ with $m\le n$, which we call the desymmetrized Robin spectrum.

### Theorem 9.1

For every $\sigma \ge 0$, the level spacing distribution for the desymmetrized Robin spectrum on the square is a delta-function at the origin.

In other words, if we denote by $\lambda _0^\sigma \le \lambda _1^\sigma \le \ldots$ the ordered (desymmetrized) Robin eigenvalues, then the cumulant of the level spacing distribution satisfies: For all $y>0$,

$$\begin{aligned} \int _0^y P(s)ds = \lim _{N\rightarrow \infty } \frac{1}{N} \#\left\{ n\le N: \frac{1}{2}\frac{{\text {area}}(\Omega )}{4\pi }(\lambda _{n+1}^\sigma -\lambda _n^\sigma ) \le y\right\} = 1. \end{aligned}$$

### Proof

The Neumann spectrum for the square consists of the numbers $$ m^2+n^2$$ (up to a multiple), with $$m,n\ge 0$$. There is a systematic double multiplicity, manifested by the symmetry $$(m,n)\mapsto (n,m)$$. We remove it by requiring $$m\le n$$. Denote the integers which are sums of two squares by

$$\begin{aligned} s_1=0<s_2=1<s_3=2<s_4=4<s_5=5<\cdots<s_{14}=25<\cdots \end{aligned}$$

We define index clusters ${\mathcal {N}}_i$ as the set of all indices of desymmetrized Neumann eigenvalues which coincide with $s_i$:

$$\begin{aligned} {\mathcal {N}}_i = \{n: \lambda _n^0=s_i\} \end{aligned}$$

For instance, $s_0=0=0^2+0^2$ has multiplicity one, and gives the index set ${\mathcal {N}}_1=\{1\}$; $s_1=1= 0^2+1^2$ has multiplicity 1 (after desymmetrization) and gives ${\mathcal {N}}_2=\{2\}$; $s_3=2=1^2+1^2$ giving ${\mathcal {N}}_3 = \{3\}$, $\ldots$ $s_{14}=25= 0^2+5^2=3^2+4^2$, ${\mathcal {N}}_{14} = \{14,15 \}$, etcetera. Then these are sets of consecutive integers which form a partition of the natural numbers $\{1,2,3,\ldots \}$, and if $i<j$ then the largest integer in ${\mathcal {N}}_i$ is smaller than the smallest integer in ${\mathcal {N}}_j$.

Denote by $\lambda _n^\sigma$ the ordered desymmetrized Robin eigenvalues: $\lambda _0^\sigma \le \lambda _1^\sigma \le \ldots$, so for $\sigma =0$ these are just the integers $s_i$ repeated with multiplicity $\#{\mathcal {N}}_i$. For each $\sigma \ge 0$, we define clusters $C_i(\sigma )$ as the set of all desymmetrized Robin eigenvalues $\lambda _n^\sigma$ with $n\in {\mathcal {N}}_i$:

$$\begin{aligned} C_i(\sigma ) = \{\lambda _n^\sigma : n\in {\mathcal {N}}_i\} . \end{aligned}$$

Now use the boundedness of the RN gaps (Theorem 1.7): $0\le \lambda _n^\sigma -\lambda _n^0\le C(\sigma )$, to deduce that the clusters have bounded diameter:

$$\begin{aligned} \mathrm{diam\,}C_i(\sigma )\le C(\sigma ) . \end{aligned}$$

If $\#{\mathcal {N}}_i=1$ then $\mathrm{diam\,}C_i(\sigma )=0$, so we may assume that $\#{\mathcal {N}}_i\ge 2$ and write

$$\begin{aligned} {\mathcal {N}}_i = \{n_-,n_-+1,\ldots , n_+\}, \quad n_+ =\max \mathcal N_i, \quad n_- =\min {\mathcal {N}}_i. \end{aligned}$$

Then

$$\begin{aligned} \begin{aligned} \mathrm{diam\,}C_i(\sigma )&= \lambda _{n_+}^\sigma -\lambda _{n_-}^\sigma \\&= (\lambda _{n_+}^\sigma -s_i)+(s_i-\lambda _{n_-}^\sigma )\\&= (\lambda _{n_+}^\sigma -\lambda _{n_+}^0)-(\lambda _{n_-}^\sigma -\lambda _{n_-}^0)\le C(\sigma )-0 = C(\sigma ) . \end{aligned} \end{aligned}$$

For the first *N* eigenvalues, the number *I* of clusters containing them is the number of the $s_i$ involved, which is at most the number of $s_i\le \lambda _N^\sigma \approx N$. A classical result of Landau [18] states that the number of integers $\le N$ which are sums of two squares is about $N/\sqrt{\log N}$, in particular^6^ is *o*(*N*). Hence

$$\begin{aligned} I\le \#\{i: s_i \ll N\} =o(N) . \end{aligned}$$

We count the number of nearest neighbour^7^ gaps $\delta _n^\sigma =\lambda _{n+1}^\sigma -\lambda _n^\sigma$ of size bigger than *y*. Of these, there are at most *I* such that $\lambda _{n+1}^\sigma$ and $\lambda _n^\sigma$ belong to different clusters, and since $I=o(N)$ their contribution is negligible. For the remaining ones, we group them by cluster to which they belong:

$$ \begin{aligned} \# \{n\le N: \delta _n^\sigma>y\} = \sum _{i=1}^I\#\left\{ n: \lambda _{n+1}^\sigma , \lambda _n^\sigma \in C_i(\sigma ) \;  \&  \; \delta _n^\sigma >y\right\} + o(N) . \end{aligned}$$

We have

$$ \begin{aligned}&\#\left\{ n: \lambda _{n+1}^\sigma , \lambda _n^\sigma \in C_i(\sigma )\;  \&  \; \delta _n^\sigma>y\right\} = \#\left\{ n\in {\mathcal {N}}_i , \; n<\max {\mathcal {N}}_i , \; \delta _n^\sigma>y\right\} \\&\qquad = \sum _{\begin{array}{c} n\in {\mathcal {N}}_i\\ n<\max {\mathcal {N}}_i \\ \delta _n^\sigma>y \end{array}} \frac{y}{y}< \sum _{\begin{array}{c} n\in {\mathcal {N}}_i\\ n<\max {\mathcal {N}}_i \\ \delta _n^\sigma >y \end{array}} \frac{\delta _n^\sigma }{y} \le \frac{1}{y} \sum _{\begin{array}{c} n\in {\mathcal {N}}_i \\ n<\max {\mathcal {N}}_i \end{array}} \delta _n^\sigma . \end{aligned}$$

The sum of nearest neighbour gaps in each cluster is

$$\begin{aligned} \sum _{\begin{array}{c} n\in {\mathcal {N}}_i \\ n<\max {\mathcal {N}}_i \end{array}} \delta _n^\sigma =\sum _{\begin{array}{c} n\in {\mathcal {N}}_i \\ n<\max {\mathcal {N}}_i \end{array}}( \lambda _{n+1}^\sigma -\lambda _n^\sigma ) =\lambda _{\max {\mathcal {N}}_i}^\sigma -\lambda _{\min \mathcal N_i}^\sigma =\mathrm{diam\,}C_i(\sigma ) \le C(\sigma ) . \end{aligned}$$

Thus we find

$$ \begin{aligned} \#\{n: \lambda _{n+1}^\sigma , \lambda _n^\sigma \in C_i(\sigma )\;  \&  \; \delta _n^\sigma >y\} \le \frac{C(\sigma )}{y} \end{aligned}$$

so that

$$\begin{aligned} \# \{n\le N: \delta _n^\sigma >y\} \le \sum _{i=1}^I \frac{C(\sigma )}{y} + o(N) = \frac{C(\sigma )}{y}I + o(N) . \end{aligned}$$

Since $I=o(N)$, and *C*, *y* are fixed, we conclude that

$$\begin{aligned} \frac{1}{N} \# \{n\le N: \delta _n^\sigma >y\} = o(1) . \end{aligned}$$

Thus the cumulant of the level spacing distribution satisfies: For all $y>0$,

$$\begin{aligned} \int _0^y P(s)ds = \lim _{N\rightarrow \infty } \frac{1}{N} \#\{n\le N: \delta _n^\sigma \le y\} = 1 \end{aligned}$$

so that *P*(*s*) is a delta function at the origin. $\quad \square$

Note that the claim is not that all gaps $\lambda _{n+1}^\sigma -\lambda _n^\sigma$ tend to zero. On the contrary, it is possible to produce thin sequences $\{n\}$ so that $\lambda _{n+1}^\sigma -\lambda _n^\sigma$ tend to infinity. Looking at the proof of Theorem 9.1, these correspond to the rare cases when $\lambda _{n}^{\sigma }$ and $\lambda _{n+1}^{\sigma }$ belong to neighboring “clusters” which are far apart from each other.

## The Unit Disk

### Upper bounds for $d_n$ via Weyl’s law

In this section we prove Theorem 1.9. We first show how to obtain upper bounds for the gaps $d_n$ from upper bounds in Weyl’s law for the Robin/Neumann problem. The result is that

#### Lemma 10.1

Let $\Omega$ be a bounded planar domain. Assume that there is some $\theta \in (0,1/2)$ so that

10.1 $$\begin{aligned} N_\sigma (x):=\#\{ \lambda _n^\sigma \le x\} = \frac{{\text {area}}(\Omega )}{4\pi }x + \frac{{\text {length}}(\partial \Omega )}{4\pi }\sqrt{x} + O_\sigma (x^{\theta }). \end{aligned}$$

and the same result holds for $\sigma =0$. Then we have

$$\begin{aligned} d_n(\sigma )\ll _\sigma n^{\theta } . \end{aligned}$$

#### Proof

We first note that (10.1) gives

10.2 $$\begin{aligned} N_0(\lambda _n^0) =n +O(n^\theta ), \end{aligned}$$

and likewise for the Robin counting function, as will be explained below. Now compare the counting functions $N_\sigma (\lambda _n^\sigma )$ and $N_0(\lambda _n^0)$ for the Robin and Neumann spectrum using (10.1) and (10.2):

$$\begin{aligned} n +O(n^\theta )= N_\sigma (\lambda _n^\sigma ) = \frac{{\text {area}}(\Omega )}{4\pi } \lambda _n^\sigma + \frac{{\text {length}}(\partial \Omega )}{4\pi }\sqrt{\lambda _n^\sigma } + O_\sigma (n^{\theta }) \end{aligned}$$

and

$$\begin{aligned} n+O(n^\theta )= N_0(\lambda _n^0) = \frac{{\text {area}}(\Omega )}{4\pi } \lambda _n^0 + \frac{{\text {length}}(\partial \Omega )}{4\pi }\sqrt{\lambda _n^0 } + O(n^{\theta }) . \end{aligned}$$

Subtracting the two gives

$$\begin{aligned} \Big ( \lambda _n^\sigma - \lambda _n^0 \Big ) \cdot \left( {\text {area}}(\Omega )+ \frac{ {\text {length}}(\partial \Omega )}{ \sqrt{\lambda _n^\sigma }+\sqrt{\lambda _n^0}}\right) = O_\sigma (n^{\theta }), \end{aligned}$$

and therefore

$$\begin{aligned} d_n(\sigma ) = \lambda _n^\sigma - \lambda _n^0 = O_\sigma (n^{\theta }). \end{aligned}$$

To show (10.2), denote $\lambda =\lambda _n^0$, and pick $\varepsilon \in (0,1)$ sufficiently small so that in the interval $[\lambda -\frac{\varepsilon }{2}, \lambda +\frac{\varepsilon }{2}]$ there are no eigenvalues other than $\lambda$, which is repeated with multiplicity $K\ge 1$. Then

$$\begin{aligned} N\left( \lambda +\frac{\varepsilon }{2}\right) -N\left( \lambda -\frac{\varepsilon }{2}\right) =K. \end{aligned}$$

On the other hand, by Weyl’s law (with $A={\text {area}}(\Omega )/4\pi$, $B={\text {length}}(\partial \Omega )/4\pi$)

$$\begin{aligned} \begin{aligned} K&=N\left( \lambda +\frac{\varepsilon }{2}\right) -N\left( \lambda -\frac{\varepsilon }{2}\right) \\&= A\left( \lambda +\frac{\varepsilon }{2}\right) +B\sqrt{\lambda +\frac{\varepsilon }{2}} +O\left( \left( \lambda +\frac{\varepsilon }{2}\right) ^\theta \right) \\&\quad - \left( A\left( \lambda -\frac{\varepsilon }{2}\right) +B\sqrt{\lambda -\frac{\varepsilon }{2}} +O\left( \left( \lambda -\frac{\varepsilon }{2}\right) ^\theta \right) \right) \\&=A\varepsilon +O\left( \frac{\varepsilon }{\sqrt{\lambda }}\right) +O(\lambda ^\theta ). \end{aligned} \end{aligned}$$

Now use $|N(\lambda _n^0) - n|\le K \ll \lambda ^\theta \ll n^\theta$ which gives (10.2). $\quad \square$

Below we implement this strategy for the disk to obtain Theorem 1.9.

### Relating Weyl’s law and a lattice point count

The eigenvalues of the Laplacian on the disk are squares of zeros of Bessel functions and understanding Weyl’s law leads to requiring knowledge of the semiclassical asymptotics of these Bessel zeros; the nature of these asymptotics leads to an exotic lattice point problem, as was exploited by Kuznetsov and Fedosov [16] and Colin De Verdière [8].

Define the domain

$$\begin{aligned} D=\left\{ \left( x,y\right) :\,x\in \left[ -1,1\right] ,\max \left( 0,-x\right) \le y\le g\left( x\right) \right\} \end{aligned}$$

where

10.3 $$\begin{aligned} g\left( x\right) =\frac{1}{\pi }\left( \sqrt{1-x^{2}}-x\arccos x\right) . \end{aligned}$$

**Fig. 6:**
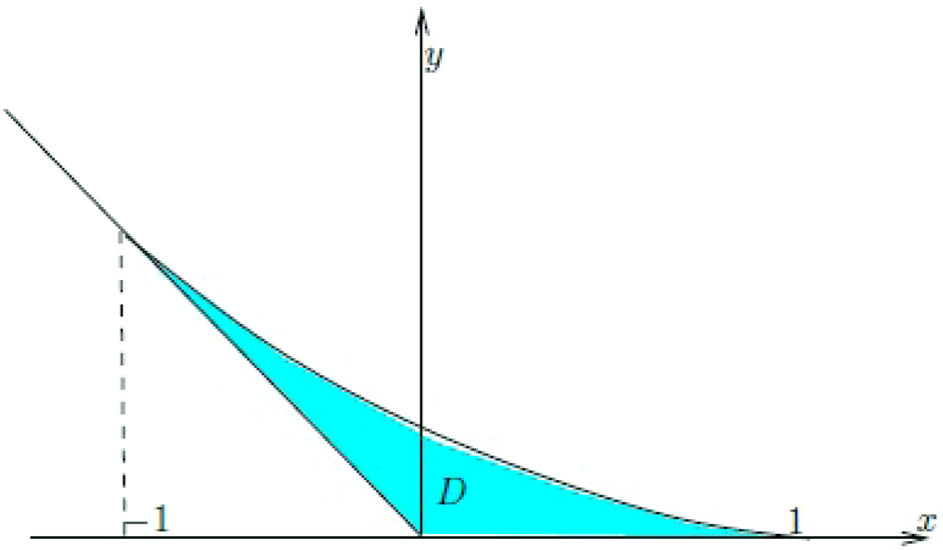
The domain *D*

Let

$$\begin{aligned} N_{D}\left( \mu \right) :=\#\left\{ \left( n,k\right) :\left( n,k+\max (0,-n)-\frac{3}{4} \right) \in \mu D\right\} \end{aligned}$$

and

$$\begin{aligned} N_{\mathrm{disk, \sigma }}\left( x \right) :=\#\left\{ \lambda _n^\sigma \le x \right\} . \end{aligned}$$

#### Proposition 10.2

Fix $$\sigma \ge 0$$. Then

$$\begin{aligned} N_{D}\left( \mu -\frac{C}{\mu ^{3/7}}\right) -C\mu ^{4/7}\le N_\mathrm{disk,\sigma }\left( \mu ^2 \right) \le N_{D}\left( \mu +\frac{C}{\mu ^{3/7}}\right) +C\mu ^{4/7} . \end{aligned}$$

The argument extends [8, Theorem 3.1], [12] (who fix a flaw in the argument of [8]) to Robin boundary conditions.

We can now prove Theorem 1.9. We use the result of [13]^8^

$$\begin{aligned} N_{D}\left( \mu \right) = {\text {area}}(D)\mu ^2+ \frac{\mu }{2}+O\Big (\mu ^{2(1/3-\delta )}\Big ) \end{aligned}$$

where $\delta =1/990$. Noting that

$$\begin{aligned} {\text {area}}(D) = \frac{{\text {area}}(\Omega )}{4\pi }=\frac{1}{4},\quad \frac{ {\text {length}}(\partial \Omega )}{4\pi } = \frac{1}{2} \end{aligned}$$

we obtain from Proposition 10.2 that

$$\begin{aligned} N_{\mathrm{disk,\sigma }}\left( x\right) = \frac{{\text {area}}(\Omega )}{4\pi } x + \frac{ {\text {length}}(\partial \Omega )}{4\pi } \sqrt{x} +O\Big (x^{1/3-\delta } \Big ) . \end{aligned}$$

Applying Lemma 10.1 gives

$$\begin{aligned} d_n(\sigma ) =O\Big ( n^{1/3-\delta } \Big ) \end{aligned}$$

which proves Theorem 1.9. $\quad \square$

### Proof of Proposition 10.2

Fix a Robin parameter $\sigma \ge 0$. Separating variables in polar coordinates $(r,\theta )$ and inserting the boundary conditions, we find a basis of eigenfunctions of the form

$$\begin{aligned} f_{n,k}(r,\theta ) = J_n(\kappa _{n,k} r)e^{i n \theta }, n\in {\mathbb {Z}}, \; k=1,2,\ldots \end{aligned}$$

with eigenvalues $\kappa _{n,k}^{2}$, where $\kappa _{n,k}$ is the *k*-th positive zero of $xJ_{n}'\left( x\right) +\sigma J_{n}\left( x\right)$. In particular, for the Neumann case ($\sigma =0$), we get zeros of the derivative $J'_n(x)$, denoted by $j'_{n,k}$; since zero is a Neumann eigenvalue we use the standard convention that $x=0$ is counted as the first zero of $J'_0(x)$.

Let

$$\begin{aligned} S=\left\{ \left( x,y\right) :y\ge \max \left( 0,-x\right) \right\} , \end{aligned}$$

and let $F:S\rightarrow {\mathbb {R}}$ be the degree 1 homogeneous function satisfying $F\equiv 1$ on the graph of *g*. Obviously,

$$\begin{aligned} F\left( n,k+\max \left( 0,-n\right) -\frac{3}{4}\right) \le \mu \Longleftrightarrow \left( n,k+\max \left( 0,-n\right) -\frac{3}{4}\right) \in \mu D; \end{aligned}$$

on the other hand, as will be shown in Lemma 10.3 below, the numbers $\kappa _{n,k}$ are well approximated by $F\left( n,k+\max \left( 0,-n\right) -\frac{3}{4}\right)$. This will give the desired connection between Weyl’s law on the disk and the lattice count problem in dilations of *D*.

#### Lemma 10.3

Fix $\sigma \ge 0$, and let $c>0$ be a constant.

1. As $n\rightarrow \infty$, uniformly for $k\le n/c$, we have

10.4 $$\begin{aligned} \kappa _{n,k}=F(n,k-\frac{3}{4})+O_{c,\sigma }\left( \frac{n^{1/3}}{k^{4/3}}\right) . \end{aligned}$$

2. As $k\rightarrow \infty$, uniformly for $\left| n\right| \le c\cdot k$, we have

10.5 $$\begin{aligned} \kappa _{n,k}=F\left( n,k+\max \left( 0,-n\right) -\frac{3}{4}\right) +O_{c,\sigma }\left( \frac{1}{k}\right) . \end{aligned}$$

The proof of Lemma 10.3 will be given in “Appendix A”.

It will be handy to derive an explicit formula for the function *F*, which we will now do. Let $\zeta =\zeta \left( z\right)$ be the solution to the differential equation

10.6 $$\begin{aligned} \left( \frac{\text {d}\zeta }{\text {d}z}\right) ^{2}=\frac{1-z^{2}}{\zeta z^{2}} \end{aligned}$$

which for $z\ge 1$ is given by

10.7 $$\begin{aligned} \frac{2}{3}\left( -\zeta \right) ^{3/2}=\sqrt{z^{2}-1}-\arccos \left( \frac{1}{z}\right) \end{aligned}$$

(see [24, Eq. 10.20.3]). The interval $z\ge 1$ is bijectively mapped to the interval $\zeta \le 0$; denote by $z=z\left( \zeta \right)$ the inverse function.

#### Lemma 10.4

For $x>0$, we have

10.8 $$\begin{aligned} F(x,y)=xz\Big (-x^{-2/3}\left( \frac{3\pi }{2}y\right) ^{2/3}\Big ). \end{aligned}$$

Additionally, for $y\ge 0$ we have $F\left( 0,y\right) =\pi y$, and for $\left( -x,y\right) \in S$ we have

10.9 $$\begin{aligned} F\left( -x,y\right) =F(x,y-x). \end{aligned}$$

#### Proof

Let $x>0$, and denote $t=\frac{F\left( x,y\right) }{x}$. Then $F\left( \frac{1}{t},\frac{y}{tx}\right) =1$ so that the point $\left( \frac{1}{t},\frac{y}{tx}\right)$ lies on the graph of *g*,  and therefore

$$\begin{aligned} \frac{y}{x}=\frac{1}{\pi }\left( \sqrt{t^{2}-1}-\arccos \left( \frac{1}{t}\right) \right) =\frac{1}{\pi }\frac{2}{3}\left( -\zeta \left( t\right) \right) ^{3/2} \end{aligned}$$

so that

$$\begin{aligned} t=z\Big (-x^{-2/3}\left( \frac{3\pi }{2}y\right) ^{2/3}\Big ). \end{aligned}$$

The other claims are also straightforward from the definitions. $\quad \square$

We proceed towards the proof of Proposition 10.2 by following the ideas of [8, Sec. 3]. Let

$$\begin{aligned} N_{D}^{1}\left( \mu \right)&=\#\left\{ \left( n,k\right) :\left( n,k+\max \left( 0,-n\right) -\frac{3}{4}\right) \in \mu D,\,\left| n\right| <c\cdot k\right\} ,\\ N_{D}^{2}\left( \mu \right)&=\#\left\{ \left( n,k\right) :\left( n,k-\frac{3}{4}\right) \in \mu D,\,n\ge c\cdot k\right\} , \end{aligned}$$

and

$$\begin{aligned} N_{\text {disk},\sigma }^{1}\left( \mu ^{2}\right)&=\#\left\{ \left( n,k\right) :\kappa _{n,k}\le \mu ,\,\left| n\right| <c\cdot k\right\} ,\\ N_{\text {disk},\sigma }^{2}\left( \mu ^{2}\right)&=\#\left\{ \left( n,k\right) :\kappa _{n,k}\le \mu ,\,n\ge c\cdot k\right\} , \end{aligned}$$

so that

$$\begin{aligned} N_{D}\left( \mu \right) =N_{D}^{1}\left( \mu \right) +2N_{D}^{2}\left( \mu \right) \end{aligned}$$

and

$$\begin{aligned} N_{\text {disk},\sigma }\left( \mu ^{2}\right) =N_{\text {disk},\sigma }^{1}\left( \mu ^{2}\right) +2N_{\text {disk},\sigma }^{2}\left( \mu ^{2}\right) , \end{aligned}$$

where we used (10.9) and the relation $\kappa _{-n,k}=\kappa _{n,k}$. We first compare $N_{D}^{1}\left( \mu \right)$ and $N_{\text {disk},\sigma }^{1}\left( \mu ^{2}\right)$:

#### Lemma 10.5

There exists a constant $C=C_{c,\sigma }>0$ such that

$$\begin{aligned} N_{D}^{1}\left( \mu -\frac{C}{\mu }\right) \le N_{\mathrm {disk},\sigma }^{1} \left( \mu ^{2}\right) \le N_{D}^{1}\left( \mu +\frac{C}{\mu } \right) . \end{aligned}$$

#### Proof

Assume that $|n|<c \cdot k$. By (10.9) and the homogeneity of *F* we have

$$\begin{aligned} F\left( n,k+\max (0,-n)-\frac{3}{4}\right) =F\left( |n|,k-\frac{3}{4}\right) =kF\left( \frac{|n|}{k},1-\frac{3}{4k}\right) , \end{aligned}$$

and since $1\ll F\left( \frac{|n|}{k},1-\frac{3}{4k}\right) \ll _c 1$, we conclude that

$$\begin{aligned} k \ll F\left( n,k+\max (0,-n)-\frac{3}{4}\right) \ll _c k.\end{aligned}$$

Hence, if $F\left( n,k+\max (0,-n)-\frac{3}{4}\right) \ge \mu$, then $k \gg _c \mu$. Combining this with Lemma 10.3, we see that

$$\begin{aligned} \begin{aligned} N_{\text {disk},\sigma }^{1}\left( \mu ^{2}\right)&\le \#\left\{ \left( n,k\right) :F(n,k+\max (0,-n )-\frac{3}{4})\le \mu +\frac{C'}{k},\,\left| n\right|<c\cdot k \right\} \\&=\#\left\{ \left( n,k\right) :F(n,k+\max \left( 0,-n\right) -\frac{3}{4})\le \mu ,\,\left| n\right|<c\cdot k\right\} \\&\quad +\#\left\{ \left( n,k\right) :\mu<F(n,k+\max \left( 0,-n\right) -\frac{3}{4})\le \mu +\frac{C'}{k},\,\left| n\right|<c\cdot k\right\} \\&\le \#\left\{ \left( n,k\right) :F(n,k+\max \left( 0,-n\right) -\frac{3}{4})\le \mu +\frac{C}{\mu },\,\left| n\right| <c\cdot k\right\} \\&=N_{D}^{1}\left( \mu +\frac{C}{\mu }\right) . \end{aligned} \end{aligned}$$

The proof of the other inequality is similar. $\quad \square$

We will now compare between $N_{D}^{2}\left( \mu \right)$ and $N_{\text {disk},\sigma }^{2}\left( \mu ^{2}\right)$. To this end, for fixed $k\ge 1$, we denote

$$\begin{aligned} N_{k}\left( \mu \right)&=\#\left\{ n:\left( n,k-\frac{3}{4}\right) \in \mu D,\,n\ge c\cdot k\right\} \\ N_{k}'\left( \mu \right)&=\#\left\{ n:\kappa _{n,k}\le \mu ,\,\,n\ge c\cdot k\right\} . \end{aligned}$$

#### Lemma 10.6

Given a sufficiently large $c>0$, there exists a constant $C=C_{c,\sigma }>0$ such that

$$\begin{aligned} N_{k}\left( \mu \right) -C\frac{\mu ^{1/3}}{k^{4/3}}-1\le N_{k}'\left( \mu \right) \le N_{k}\left( \mu \right) +C\frac{\mu ^{1/3}}{k^{4/3}}+1. \end{aligned}$$

#### Proof

Let

$$\begin{aligned} A_{k}\left( \mu \right) :=\#\left\{ n:\,\mu <F\left( n,k-\frac{3}{4}\right) \le \mu +C'\frac{\mu ^{1/3}}{k^{4/3}},\,n\ge c\cdot k\right\} , \end{aligned}$$

and recall the inequality (see (A.1)) $n \le j'_{n,k } \le \kappa _{n,k}$, so in particular if $\kappa _{n,k} \le \mu$, then $n\le \mu$. Thus, Lemma 10.3 gives

$$\begin{aligned} N_{k}'\left( \mu \right)&\le \#\left\{ n:F \left( n,k-\frac{3}{4}\right) \le \mu +C'\frac{n^{1/3}}{k^{4/3}},\,\mu \ge n\ge c\cdot k\right\} \\&\le \#\left\{ n:F \left( n,k-\frac{3}{4} \right) \le \mu ,\,n\ge c\cdot k\right\} +A_{k}\left( \mu \right) \\&=N_{k}\left( \mu \right) +A_{k}\left( \mu \right) . \end{aligned}$$

When $x\ge c \cdot k$, we have $F\left( x,k-\dfrac{3}{4}\right) =xF\left( 1,\dfrac{k-3/4}{x}\right)$, and therefore (note that $F(1,y)\ge 1$ for all $y\ge 0$)

$$\begin{aligned} F_x\left( x,k-\dfrac{3}{4}\right) = F\left( 1,\dfrac{k-3/4}{x}\right) -\dfrac{k-3/4}{x}F_y\left( 1,\dfrac{k-3/4}{x}\right) \gg 1 \end{aligned}$$

when *c* is taken sufficiently large. In particular, ${\tilde{F}}(x) := F(x,k-\dfrac{3}{4})$ is strictly increasing for $x\ge c \cdot k$, and so $A_k(\mu )$ is bounded above by the number of integer points in the interval

$$\begin{aligned} I := \left[ {\tilde{F}}^{-1}\left( \max (\mu ,{\tilde{F}}(c\cdot k))\right) , {\tilde{F}}^{-1}\left( \mu +C'\frac{\mu ^{1/3}}{k^{4/3}}\right) \right] , \end{aligned}$$

which in turn is bounded above by $\mathrm {length}(I) + 1$; by the mean value theorem, keeping in mind that $({\tilde{F}}^{-1})_x = {\tilde{F}}_x^{-1},$ we conclude that

$$\begin{aligned} \mathrm {length}(I) \le C'\frac{\mu ^{1/3}}{k^{4/3}} \cdot \max _{x\in I} \dfrac{1}{{\tilde{F}}_x(x)} \le C \frac{\mu ^{1/3}}{k^{4/3}}. \end{aligned}$$

The proof of the other inequality is similar. $\quad \square$

#### Remark 10.7

The $+1$ factor was missing in [8].

For large values of *k* we will use the following estimate:

#### Lemma 10.8

There exists a constant $C=C_{c,\sigma }>0$ such that for $k>\mu ^{4/7}$, we have

$$\begin{aligned} N_{k}\left( \mu -\frac{C}{\mu ^{3/7}}\right) \le N_{k}'\left( \mu \right) \le N_{k}\left( \mu +\frac{C}{\mu ^{3/7}}\right) . \end{aligned}$$

#### Proof

By Lemma 10.3,

$$\begin{aligned} N_{k}'\left( \mu \right) \le \#\left\{ n:F\left( n,k-\frac{3}{4}\right) \le \mu +\frac{C}{\mu ^{3/7}},\,n\ge c\cdot k\right\} =N_{k}\left( \mu +\frac{C}{\mu ^{3/7}}\right) \end{aligned}$$

and likewise

$$\begin{aligned} N_{k}'\left( \mu \right) \ge N_{k}\left( \mu -\frac{C}{\mu ^{3/7}}\right) . \end{aligned}$$

$$\square $$

#### Proof of Proposition 10.2

By Lemma 10.6 (applied for $k\le \mu ^{4/7}$) and Lemma 10.8 (applied for $k>\mu ^{4/7}$), we get that

$$\begin{aligned} N_{\text {disk},\sigma }^{2}\left( \mu ^{2}\right)= & {} \sum _{k\ge 1}N_{k}'\left( \mu \right) \\\le & {} \sum _{k\ge 1}N_{k}\left( \mu +\frac{C}{\mu ^{3/7}}\right) +C\mu ^{4/7} =N_{D}^{2}\left( \mu +\frac{C}{\mu ^{3/7}}\right) +C\mu ^{4/7} \end{aligned}$$

and likewise

$$\begin{aligned} N_{\text {disk},\sigma }^{2}\left( \mu ^{2}\right) \ge N_{D}^{2}\left( \mu -\frac{C}{\mu ^{3/7}}\right) -C\mu ^{4/7}. \end{aligned}$$

This, together with Lemma 10.5 gives the claim. $\quad \square$

## Appendix A. Proof of Lemma 10.3

The goal of this appendix is to prove the asymptotic formulas (10.4) and (10.5) for the zeros $\kappa _{n,k}$ of $xJ_{n}'\left( x\right) +\sigma J_{n}\left( x\right)$ where $\sigma \ge 0.$ More generally, we will work with Bessel functions $J_{\nu }\left( x\right)$ of real order $\nu$. Many properties of the zeros $\kappa _{\nu ,k}$ are well-known, e.g. for all $\sigma >0$, $\nu \ge 0$ and $k\ge 1$ we have (see e.g. [33, Eq. (III.6)])

A.1 $$\begin{aligned} \nu \le j'_{\nu ,k}<\kappa _{\nu ,k}<j_{\nu ,k} \end{aligned}$$

where $j_{\nu ,k}$ (resp. $j'_{\nu ,k}$) is the *k*-th positive zero of $J_{\nu }\left( x\right)$ (resp. $J'_{\nu }\left( x\right)$, with the convention that $x=0$ is counted as the first zero of $J'_0(x)$); for $\sigma \ge 0$ and fixed $\nu \ge 0$ we have the asymptotic formula [33, Eq. (IV.9)]

$$\begin{aligned} \kappa _{\nu ,k}=j'_{\nu ,k}+\frac{\sigma }{j'_{\nu ,k}}+\frac{-\frac{1}{3}\sigma ^{3} -\frac{1}{2}\sigma ^{2}+\nu ^{2}\sigma }{\left( j'_{\nu ,k}\right) ^{3}}+O_{\sigma }\left( \left( j'_{\nu ,k}\right) ^{-5} \right) \qquad \left( k\rightarrow \infty \right) . \end{aligned}$$

Recall the function $\zeta \left( z\right)$ defined above by (10.6) which satisfies (10.7) for $z\ge 1$, with an inverse $z\left( \zeta \right)$. Denote $h\left( \zeta \right) =\left( \frac{4\zeta }{1-z^{2}}\right) ^{1/4}$. We have the following asymptotic expansion for $J_{\nu }\left( \nu z\right)$ as $\nu \rightarrow \infty$ [24, Eq. 10.20.4]

A.2 $$\begin{aligned} J_{\nu }\left( \nu z\right) \sim h\left( \zeta \right) \left[ \frac{\text {Ai}\left( \nu ^{2/3}\zeta \right) }{\nu ^{1/3}}\sum _{j=0}^{\infty }\frac{A_{j}\left( \zeta \right) }{\nu ^{2j}}+\frac{\text {Ai}'\left( \nu ^{2/3}\zeta \right) }{\nu ^{5/3}}\sum _{j=0}^{\infty }\frac{B_{j}\left( \zeta \right) }{\nu ^{2j}}\right] \end{aligned}$$

which holds uniformly for $$ z>0, $$ where $$\text {Ai }\left( z\right) $$ is the Airy function, and the coefficients $$A_{j}\left( \zeta \right) $$ and $$B_{j}\left( \zeta \right) $$ are given by [24, Eq. 10.2.10, 10.20.11] and the remark following these equations. Likewise, we have the asymptotic expansion [24, Eq. 10.20.7]

A.3 $$\begin{aligned} J_{\nu }'\left( \nu z\right) \sim -\frac{2}{zh\left( \zeta \right) }\left[ \frac{\text {Ai}\left( \nu ^{2/3}\zeta \right) }{\nu ^{4/3}}\sum _{j=0}^{\infty }\frac{C_{j}\left( \zeta \right) }{\nu ^{2j}}+\frac{\text {Ai}'\left( \nu ^{2/3}\zeta \right) }{\nu ^{2/3}}\sum _{j=0}^{\infty }\frac{D_{j}\left( \zeta \right) }{\nu ^{2j}}\right] \end{aligned}$$

uniformly for $$ z>0, $$ where the coefficients $$C_{j}\left( \zeta \right) $$ and $$D_{j}\left( \zeta \right) $$ are given by [24, Eq. 10.2.12, 10.20.13] and the remark which follows them. Each of the coefficients $$A_{j}\left( \zeta \right) ,B_{j}\left( \zeta \right) ,C_{j}\left( \zeta \right) ,D_{j}\left( \zeta \right) $$, $$j=0,1,2,\ldots $$ is bounded near $$\zeta =0$$; we have $$A_{0}\left( \zeta \right) =D_{0}\left( \zeta \right) =1$$.

For the Robin parameter $\sigma \ge 0$, if we denote $B_{-1}\left( \zeta \right) =0$ and let

$$\begin{aligned} \alpha _{j}^{\sigma }\left( \zeta \right)&:=C_{j}\left( \zeta \right) -\frac{\sigma A_{j}\left( \zeta \right) h^{2}\left( \zeta \right) }{2}\\ \beta _{j}^{\sigma }\left( \zeta \right)&:=D_{j}\left( \zeta \right) -\frac{\sigma B_{j-1}\left( \zeta \right) h^{2}\left( \zeta \right) }{2}, \end{aligned}$$

then (A.2) and (A.3) give

$$\begin{aligned} \phi _{\nu }\left( \nu z\right)&:=J_{\nu }'\left( \nu z\right) +\frac{\sigma }{\nu z}J_{\nu }\left( \nu z\right) \\&\sim \frac{-2}{zh\left( \zeta \right) }\left[ \frac{\text {Ai}'\left( \nu ^{2/3}\zeta \right) }{\nu ^{2/3}}\sum _{j=0}^{\infty }\frac{\beta _{j}^{\sigma }\left( z\right) }{\nu ^{2j}}+\frac{\text {Ai}\left( \nu ^{2/3}\zeta \right) }{\nu ^{4/3}}\sum _{j=0}^{\infty }\frac{\alpha _{j}^{\sigma }\left( z\right) }{\nu ^{2j}}\right] \end{aligned}$$

uniformly for $z>0.$ Note that $\alpha _{0}^{\sigma }\left( \zeta \right) =C_{0}\left( \zeta \right) -\frac{\sigma h^{2}\left( \zeta \right) }{2}$. Using the derivation of [25, p. 345] with $\alpha _{j}^{\sigma }$, $\beta _{j}^{\sigma }$ instead of $C_{j}\left( \zeta \right)$, $D_{j}\left( \zeta \right)$ (the latter were used to establish the asymptotic expansion of the zeros of $J_{\nu }'\left( z\right)$ corresponding to $\sigma =0$), we get the following *uniform* asymptotic formula for $\kappa _{\nu ,k}$ as $\nu \rightarrow \infty$ :

### Lemma A.1

Fix $\sigma \ge 0$, let $a_{k}'$ be the *k*-th zero of $\mathrm {Ai}'\left( z\right)$ (all of these zeros are real and negative), and let $\zeta =\zeta _{\nu ,k}=\nu ^{-2/3}a_{k}'$. Then in the above notation, uniformly for $k\ge 1$, we have

A.4 $$\begin{aligned} \kappa _{\nu ,k}=\nu z\left( \zeta \right) -\frac{z'\left( \zeta \right) \left( C_{0}\left( \zeta \right) -\frac{\sigma h^{2}\left( \zeta \right) }{2}\right) }{\zeta \nu }+O_{\sigma }\left( \frac{1}{\nu }\right) . \end{aligned}$$

In particular, for $\sigma =0$ we reconstructed the formula [24, Eq. 10.21.43]

$$\begin{aligned} j'_{\nu ,k}=\nu z\left( \zeta \right) -\frac{z'\left( \zeta \right) C_{0}\left( \zeta \right) }{\zeta \nu }+O\left( \frac{1}{\nu }\right) \end{aligned}$$

uniformly for $k\ge 1$ (note the identity $z'\left( \zeta \right) =-\frac{z\left( \zeta \right) h^{2}\left( \zeta \right) }{2}$). We remark that the secondary term in (A.4) is necessary because of the $\zeta$ factor in the denominator which may be as small as $\nu ^{-2/3}$ when *k* is small. This phenomenon does not occur for the zeros of $J_{\nu }\left( z\right)$ (see [25, Sec. 7]), which satisfy the more compact uniform expansion

$$\begin{aligned} j_{\nu ,k}=\nu z\left( \zeta \right) +O\left( \frac{1}{\nu }\right) \end{aligned}$$

where $\zeta =\nu ^{-2/3}a_{k}$ and $a_{k}$ is the *k*-th zero of $\text {Ai}\left( z\right)$ [24, Eq. 10.21.41].

Recall that the zeros $a_{k}'$ of $\text {Ai}'\left( z\right)$ satisfy the asymptotic formula [24, Eq. 9.9.8]

A.5 $$\begin{aligned} a_{k}'=-\left[ \frac{3\pi }{2}\left( k-\frac{3}{4}\right) \right] ^{2/3}+O\left( k^{-4/3}\right) . \end{aligned}$$

### Proof of Lemma 10.3, first part

Assume that $k\le \nu /c$, where $\nu \rightarrow \infty$. The functions $z'\left( \zeta \right)$ and $h\left( \zeta \right)$ are bounded near $\zeta =0$, and therefore inserting (A.5) into (A.4) gives

$$\begin{aligned} \nu z\left( \zeta \right) =\nu z\left( -\nu ^{-2/3}\left[ \frac{3\pi }{2}\left( k-\frac{3}{4}\right) \right] ^{2/3}\right) +O_{c,\sigma } \left( \frac{\nu ^{1/3}}{k^{4/3}}\right) \end{aligned}$$

and

$$\begin{aligned} \frac{z'\left( \zeta \right) \left( C_{0}\left( \zeta \right) -\frac{\sigma h^{2}\left( \zeta \right) }{2}\right) }{\zeta \nu }\ll _{c,\sigma }\frac{1}{\nu ^{1/3}k^{2/3}}\ll _c\frac{\nu ^{1/3}}{k^{4/3}}. \end{aligned}$$

Also note that $\frac{1}{\nu }\ll _c\frac{\nu ^{1/3}}{k^{4/3}}$ when $k\le \nu$/c. By (10.8) we have

$$\begin{aligned} z\left( -\nu ^{-2/3}\left[ \frac{3\pi }{2}\left( k-\frac{3}{4}\right) \right] ^{2/3}\right) =F\left( \nu ,k-\frac{3}{4}\right) , \end{aligned}$$

and therefore the above estimates yield

A.6 $$\begin{aligned} \kappa _{\nu ,k}=F\left( \nu ,k-\frac{3}{4}\right) +O_{c,\sigma }\left( \frac{\nu ^{1/3}}{k^{4/3}}\right) \end{aligned}$$

which gives (10.4). $$\quad \square $$

In order to prove the second part of Lemma 10.3, we require the following lemma. Recall the function $g\left( x\right)$ defined in (10.3).

### Lemma A.2

Fix $\sigma \ge 0$, and let $C>0$ be a constant. Let $\phi _{\nu }\left( x\right) :=J_{\nu }'\left( x\right) +\frac{\sigma }{x}J_{\nu }\left( x\right)$. As $x\rightarrow \infty$, uniformly for $0\le \nu \le x/\left( 1+C\right)$, we have

A.7 $$\begin{aligned} \phi _{\nu }\left( x\right) =-\left( \frac{2}{\pi }\right) ^{1/2}\left( x^{2}-\nu ^{2}\right) ^{1/4}x^{-1}\left( \sin \left( \pi xg\left( \nu /x\right) -\frac{\pi }{4}\right) +O_{C,\sigma }\left( x^{-1}\right) \right) .\nonumber \\ \end{aligned}$$

### Proof

We use the standard integral representation [24, Eq. 10.9.6]

A.8 $$\begin{aligned} J_{\nu }\left( x\right) =\frac{1}{\pi }\int _{0}^{\pi }\cos \left( x\sin t-\nu t\right) \,dt-\frac{\sin \left( \nu \pi \right) }{\pi }\int _{0}^{\infty }e^{-x\sinh t-\nu t}\,dt \end{aligned}$$

(for integer $\nu$ the second integral in (A.8) vanishes). Assume that $x\ge \left( 1+C\right) \nu \ge 0$, and denote $r=\nu /x\le \frac{1}{1+C}<1$. The first integral in (A.8) is equal to the real part of

$$\begin{aligned} {\mathcal {I}}_{r}^{1}\left( x\right) :=\frac{1}{\pi }\int _{0}^{\pi }e^{ix\left( \sin t-rt\right) }\,dt. \end{aligned}$$

By the method of stationary phase [24, Eq. 2.3.23], we have the asymptotics:

$$\begin{aligned} {\mathcal {I}}_{r}^{1}\left( x\right)&=e^{\pi i\left( xg\left( r\right) -\frac{1}{4}\right) }\left( \frac{2}{\pi \sqrt{1-r^{2}}x}\right) ^{1/2}\\&\quad +e^{-ir\pi x}\frac{i}{\pi \left( 1+r\right) x}+\frac{i}{\pi \left( 1-r\right) x}+O_{C}\left( x^{-3/2}\right) . \end{aligned}$$

Hence

$$\begin{aligned} \text {Re}\left( {\mathcal {I}}_{r}^{1}\left( x\right) \right)&=\cos \left( \pi xg\left( r\right) -\frac{\pi }{4}\right) \left( \frac{2}{\pi \sqrt{1-r^{2}}x}\right) ^{1/2}\\&\quad +\frac{\sin \left( \pi rx\right) }{\pi \left( 1+r\right) x}+O_{C}\left( x^{-3/2}\right) . \end{aligned}$$

The second integral in (A.8) is equal to

$$\begin{aligned} {\mathcal {I}}_{r}^{2}\left( x\right) :=\frac{\sin \left( \pi rx\right) }{\pi }\int _{0}^{\infty }e^{-x\left( \sinh t+rt\right) }\,dt \end{aligned}$$

and can be evaluated by the Laplace method [24, Eq. 2.3.15]:

$$\begin{aligned} {\mathcal {I}}_{r}^{2}\left( x\right) =\frac{\sin \left( \pi rx\right) }{\pi \left( 1+r\right) x}+O_{C}\left( x^{-2}\right) . \end{aligned}$$

We obtain

A.9 $$\begin{aligned} J_{\nu }\left( x\right) =\left( \frac{2}{\pi }\right) ^{1/2}\left( x^{2}-\nu ^{2}\right) ^{-1/4}\left( \cos \left( \pi xg\left( \nu /x\right) -\frac{\pi }{4}\right) +O_{C}\left( x^{-1}\right) \right) ; \end{aligned}$$

a similar procedure gives

A.10 $$\begin{aligned} J_{\nu }'\left( x\right) =-\left( \frac{2}{\pi }\right) ^{1/2}\left( x^{2}-\nu ^{2}\right) ^{1/4}x^{-1}\left( \sin \left( \pi xg\left( \nu /x\right) -\frac{\pi }{4}\right) +O_{C}\left( x^{-1}\right) \right) .\nonumber \\ \end{aligned}$$

The formula (A.7) now follows upon combining (A.9) and (A.10). $\quad \square$

### Proof of Lemma 10.3, second part

Let $0\le \nu \le c\cdot k,$ where $k\rightarrow \infty$. Clearly, the condition $0\le \nu \le c\cdot k$ implies that $\kappa _{\nu ,k}\ge \left( 1+C\right) \nu \ge 0$ for some constant $C=C\left( c\right) >0$ and that $\kappa _{\nu ,k} \asymp _c k$ (e.g. by the analogous well-known inequalities for the Bessel zeros $j_{\nu ,k}$, see (5.3) in [9], together with (A.1)). By Lemma A.2, we have

$$\begin{aligned} \sin \left( \pi \kappa _{\nu ,k} g\left( \nu /\kappa _{\nu ,k}\right) -\frac{\pi }{4}\right) =O_{c, \sigma }\left( k^{-1}\right) \end{aligned}$$

so there exists an integer *m* such that

A.11 $$\begin{aligned} \kappa _{\nu ,k}g\left( \nu /\kappa _{\nu ,k}\right) =m-\frac{3}{4}+O_{c,\sigma }\left( k^{-1}\right) . \end{aligned}$$

This in particular gives $m \asymp _{c,\sigma } \kappa _{\nu ,k}$. We have 1 $\ll F_{y} \ll _{c,\sigma } 1$ when $y\gg _{c,\sigma } x$, as can be seen by differentiating (10.8) and combining with (10.6). This, together with the equality $\kappa _{\nu ,k} = F(\nu , \kappa _{\nu ,k}g(\nu / \kappa _{\nu ,k}))$ and (A.11) gives

$$\begin{aligned} \kappa _{\nu ,k}=F\left( \nu ,m-\frac{3}{4}+O_{c,\sigma }\left( k^{-1}\right) \right) =F\left( \nu ,m-\frac{3}{4}\right) +O_{c,\sigma }\left( k^{-1}\right) . \end{aligned}$$

We will now show that $m=k$: indeed, fix $k, \nu$ and the corresponding *m*, and assume that $\nu '$ is close to $\nu$, so there exists an integer $m'$ such that

$$\begin{aligned} \kappa _{\nu ',k}=F\left( \nu ',m'-\frac{3}{4}\right) +O_{c,\sigma }\left( k^{-1}\right) . \end{aligned}$$

By the mean value theorem

A.12 $$\begin{aligned} |m-m'|&\ll \left| F\left( \nu ',m-\frac{3}{4}\right) - F\left( \nu ',m'-\frac{3}{4}\right) \right| \nonumber \\&\le \left| F\left( \nu ',m-\frac{3}{4}\right) - F\left( \nu ,m-\frac{3}{4}\right) \right| + |\kappa _{\nu ',k}-\kappa _{\nu ,k}| \nonumber \\&\quad +O_{c,\sigma }\left( k^{-1}\right) . \end{aligned}$$

Note that $\kappa _{\nu ',k}$ is a continuous function of $\nu '\ge 0$ (in fact, it is differentiable in $\nu '$ in this regime, see e.g. [19]), and so is $F\left( \nu ',m-\frac{3}{4}\right)$ as a function of $\nu '$. Therefore the right-hand-side of (A.12) can be made arbitrarily small when *k* is sufficiently large and $\nu '$ is sufficiently close to $\nu$, and hence $m'=m$. We see that map $\nu \mapsto \kappa _{\nu ,k} \mapsto m$ is well-defined and it is locally constant and hence constant for $0 \le \nu \le c \cdot k$. But we know by (A.6) that for $\nu \asymp k$ we have $m=k$; hence $m=k$ for any $0 \le \nu \le c \cdot k$. This gives (10.5) when $n\ge 0;$ for $n<0$, (10.4) follows from the relations $\kappa _{-n,k}=\kappa _{n,k}$ and (10.9).$\quad \square$

## Footnotes

1 Here and in the sequel we use $A\ll B$ as an alternative to $A=O(B)$.

2 Their Proposition 2.4 is stated only for the Neumann case, but as is pointed out in Remark 2.7, the proof applies to Robin case as well, uniformly in $\sigma \ge 0$; and they attribute it to Tataru [34, Theorem 3]. Note that their convention for the normal derivative is different than ours.

3 If we also allow negative Robin constant $\sigma <0$, we may have a finite number of negative eigenvalues and this part of the argument would not work for these.

4 [1, Lemma 2.11] allows $\Omega {\subset } {\mathbb {R}}^N$ to be any bounded Lipschitz domain and takes $\sigma \equiv 1$.

5 Of course, in this case it directly follows from the secular equation (8.1).

6 This is much easier to show using a sieve.

7 For simplicity we replace $\frac{1}{2} \frac{{\text {area}}(\Omega )}{4\pi }$ by 1, that is we don’t bother normalizing so as to have mean gap unity; the result is independent of this normalization.

8 They treat the shifted lattice ${\mathbb {Z}}^2-(0,\frac{1}{4})$ but as they say [13, Remark 6.5], the arguments also work for the shift by $(0,\frac{3}{4})$. See [12] for an a further improvement in the Dirichlet case to $131/416=1/3-23/1248= 0.314904$.
